# Predictive value of admission red cell distribution width-to-platelet ratio for 30-day death in patients with spontaneous intracerebral hemorrhage: an analysis of the MIMIC database

**DOI:** 10.3389/fneur.2023.1221335

**Published:** 2023-10-18

**Authors:** Hanbai Liang, Ping Liu, Lei Guo, Jie Feng, Cheng Yin, Dongdong Zhao, Longyi Chen

**Affiliations:** Department of Neurosurgery, Sichuan Provincial People's Hospital, University of Electronic Science and Technology of China, Chengdu, China

**Keywords:** spontaneous intracerebral hemorrhage, red cell distribution width to platelet ratio, 30-day death, prediction, mimic

## Abstract

**Aim:**

Prognostic assessment plays an important role in the effective management of patients with spontaneous intracerebral hemorrhage (ICH). The study aimed to investigate whether elevated red cell distribution width-to-platelet ratio (RPR) at admission was related to 30-day death in patients with spontaneous intracerebral hemorrhage (ICH).

**Methods:**

This retrospective cohort study included 2,823 adult patients with ICH from the Multiparameter Intelligent Monitoring in Intensive Care (MIMIC) III and IV databases between 2001 and 2019. The Cox proportional hazard model was utilized to evaluate the relationship between RPR levels and 30-day death risk. The area under receiver-operating characteristic curve (AUC) was used to assess the predictive ability of RPR for 30-day death in patients with ICH.

**Results:**

At the end of the 30-day follow-up, 799 (28.30%) patients died, and the median RPR level was 0.066 (0.053, 0.087). After adjusting for confounders, the tertile 3 of RPR levels [hazard ratio (HR) = 1.37, 95% confidence interval (CI): 1.15–1.64] were associated with a higher risk of 30-day death in patients with ICH compared with tertile 1. In the stratified analyses, elevated RPR levels were found to be associated with an increased risk of 30-day death in patients aged <65 years (HR = 1.77, 95%CI: 1.29–2.43), aged ≥65 years (HR = 1.30, 95%CI: 1.05–1.61), with Glasgow Coma Score (GCS) <14 (HR = 1.65, 95%CI: 1.27–2.14), with Charlson comorbidity index (CCI) ≥4 (HR = 1.45, 95%CI: 1.17–1.80), with (HR = 1.66, 95%CI: 1.13–2.43) or without sepsis (HR = 1.32, 95%CI: 1.08–1.61), and female patients (HR = 1.75, 95%CI: 1.35–2.26) but not in male patients (*P* = 0.139) and patients with GCS ≥14 (*P* = 0.058) or CCI <4 (*P* = 0.188). The AUC for RPR to predict 30-day death in patients with ICH was 0.795 (95%CI: 0.763–0.828) in the testing set, indicating a good predictive ability.

**Conclusion:**

Elevated RPR levels were correlated with an increased risk of 30-day death in patients with ICH, and RPP levels showed good predictive ability for 30-day death.

## Introduction

Spontaneous intracerebral hemorrhage (ICH) is a global, life-threatening disease with a poor prognosis and few proven treatments (1). Due to the non-traumatic rupture of intracranial vessels, blood flows into the brain parenchyma or ventricles and forms hematomas, causing neuronal and glial damage, which in turn causes an inflammatory response (2). ICH accounts for approximately 30% of all acute strokes and is related to high mortality and morbidity (3). In addition, more than 30% of deaths in patients with ICH occur within 30 days of the hemorrhage (4). The effective management of ICH patients includes close monitoring and treatment of blood pressure, seizures, elevated intracranial pressure, and reversal of anticoagulation and antiplatelet medications (2, 5). Reliable tools for patient prognostic assessment are essential for the treatment and management of the disease.

The inflammatory response is closely associated with intracerebral blood extravasation in patients with ICH (6, 7). Inflammatory changes in the tissue surrounding the hematoma led to an immune response, followed by the activation of microglia and cytokine release (8–10). A systematic review summarized the prognostic role of serum biomarkers, such as neutrophil–lymphocyte ratio, S100 calcium-binding protein B, and thioredoxin, in patients with ICH (11). However, there is still a lack of routine blood indicators with sufficient clinical evidence to evaluate the prognosis of ICH patients. Several studies have reported that red cell distribution width (RDW)-to-platelet ratio (RPR) correlated with the prognosis of various diseases such as hepatic fibrosis (12), sepsis (13), and acute traumatic brain injury (14). RDW and platelet represent the heterogeneity of circulating red blood cells and the pathophysiology of hemostasis, respectively. Recently, Lehmann et al. investigated the association between RPR and 90-day mortality in patients with ICH (15). However, their study was limited by the small sample size, and the association between RPR and death in patients with ICH across age and disease severity was unknown. Furthermore, the predictive ability of RPR for death in ICH patients is unclear.

We aimed to investigate the association between RPR levels and 30-day death in patients with ICH based on a large sample of the database. In addition, the predictive ability of RPR for 30-day death in patients with ICH was evaluated.

## Methods

### Data source and study population

All data in this retrospective cohort study were extracted from the Multiparameter Intelligent Monitoring in Intensive Care III and IV (MIMIC-III and -IV) database between 2001 and 2019 (https://mimic.mit.edu/docs/iii/). MIMIC-III is a large, freely available database that includes non-identifiable health data related to more than 40,000 patients admitted to the intensive care unit (ICU) at Beth Israel Deaconess Medical Center between 2001 and 2012. MIMIC-IV is an updated version of MIMIC-III and currently contains data on patients admitted to the ICU at the Beth Israel Deaconess Medical Center between 2008 and 2019. The MIMIC database includes information on demographic, vital sign measurements, laboratory testing, procedures, medications, caregiver notes, imaging reports, and mortality. To access the databases, the author completed the online training for the Collaborative Institutional Training Initiative program of the National Institutes of Health. International Classification of Diseases, Ninth and Ten Revision (ICD-9 and ICD-10) codes [ICD-9 (431), ICD-10 (I610-I619)] were utilized to identify patients with ICH. Patients diagnosed with ICH were potentially eligible for inclusion. The excluded criteria were as follows: (1) patients aged <18 years or ≥90 years; (2) patients admitted to the ICU for less than 24 h; (3) patients with missing important variables such as RDW and platelet. The MIMIC database was approved by the institution review boards of the Massachusetts Institute of Technology and Beth Israel Deaconess Medical Center. This study was exempted from ethical review by Sichuan Provincial People's Hospital because it used de-identified data derived from a public MIMIC database.

### Outcomes

The study outcome was 30-day death, which was defined as death within 30 days after the bleeding event. Complete survival data were recorded up to a 30-day follow-up. For patients with more than one ICU admission, only data from the first ICU admission were selected for analysis. RPR was calculated as RPR = red blood cell distribution width (%)/platelet count (K/uL). Red blood cell distribution width and platelet count were used as measured at the time of patient admission. RPR value was divided into three categories based on the tri-sectional quantile: tertile 1, <0.057; tertile 2, 0.057-0.078; and tertile 3, >0.078.

### Data collection

Demographic information, vital sign measurements, laboratory testing data, medication and disease information, and scores were collected from the patient's medical records including age, sex (female and male), race (white, black, others, and unknown), insurance (Medicare, private, and others), admission type (emergency and non-emergency), ICU type [medical ICU (MICU), surgical ICU (SICU), and others], ventilation, vasopressor, renal replacement therapy, congestive heart failure, sepsis, atrial fibrillation, hypertension, malignant cancer, diabetes, systolic blood pressure (SBP), diastolic blood pressure (DBP), respiratory rate, heart rate, temperature, saturation of peripheral oxygen (SPO_2_), Simplified Acute Physiology Score (SAPS) II, Sequential Organ Failure Assessment (SOFA) score, quick SOFA (qSOFA) score, Glasgow Coma Score (GCS), Charlson comorbidity index (CCI), white blood cell (WBC), hemoglobin, hematocrit, creatinine, international normalized ratio (INR), prothrombin time, blood urea nitrogen, glucose, bicarbonate, sodium, potassium, chloride, urine output, mannitol, anticoagulation, blood transfusion, surgery (craniotomy, minimally invasive surgery, and no surgery), neurodegeneration, length of hospital stay, ICU stay, ICU status (ICU death, ICU discharge, and ICU readmission), survival time within 30-day, survival status within 30-day, and RPR value.

### Management of missing data

There were no missing data for 30-day survival and RPR value. Variables with more than 20% missing values were excluded. Variables with less than 20% missing data were interpolated using the random forest interpolation method (n_estimators = 500). Sensitivity analysis was performed by analyzing the differences between the data before and after interpolation.

### Statistical analysis

Continuous variables were expressed as mean and standard deviation (mean ± SD) or median and quartiles [M (Q1, Q3)] and compared using Student's *t*-test or the Wilcoxon rank-sum test. Categorical variables were expressed as number and percentage [*n* (%)] and compared using the chi-square test or Fisher's exact test.

The univariate Cox proportional hazard model was used to screen variables that may be associated with 30-day death in patients with ICH. Variables with a significant statistical difference in univariate analysis were screened by stepwise regression with bidirectional elimination, and the final screened variables were included as confounders in the multivariate Cox proportional hazard model. The association between RPR and the risk of 30-day death in ICH patients was analyzed by a multivariate Cox proportional hazard model and presented as hazard risk (HR) with a 95% confidence interval (CI). The concordance index (C-index) was used to evaluate discriminative ability. Kaplan–Meier (K-M) survival curves were plotted for ICH patients with different RPR values. The association between RPR levels and 30-day death in ICH patients was further analyzed based on age (<65 and ≥65 years), sex (female and male), GCS score (<14 and ≥14), CCI score (<4 and ≥4), and sepsis (yes and no). To compare the predictive ability of RPR, SOFA, SAPS II, and qSOFA for 30-day death in patients with ICH, all patients were randomly divided into a training set and a testing set with a ratio of 7:3. The characteristics of patients in the training set and testing set were shown in Supplementary Table 1. Receiver operating characteristic (ROC) curves for the prediction of death in patients with ICH by different tools were constructed, and the area under the curve (AUC) was calculated. The Delong test was utilized to compare the difference in AUC between tools. C-index and AUC values >0.7 indicate a reasonable estimate.

All statistical analyses were performed using SAS 9.4 software (SAS Institute Inc., Cary, NC, USA) and R 4.2.0 software (Institute for Statistics and Mathematics, Vienna, Austria). A two-sided *P*-value < 0.05 was considered to be statistically significant.

## Results

### Patient characteristics

**Figure 1:**
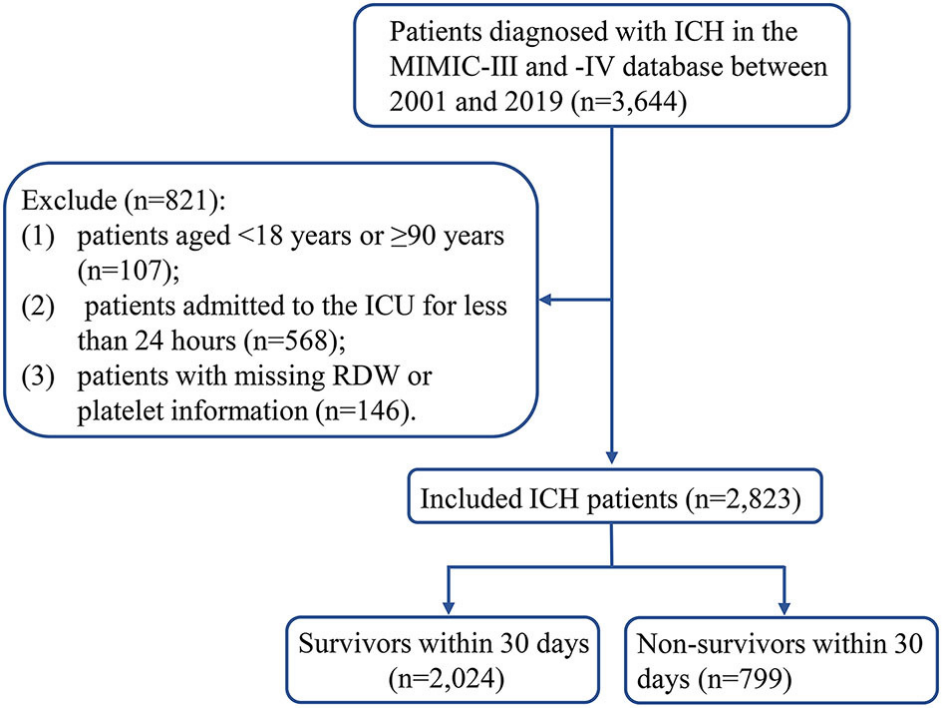
Flow chart of the study population. ICH, spontaneous intracerebral hemorrhage; MIMIC, Multiparameter Intelligent Monitoring in Intensive Care; ICU, intensive care unit; RDW, red cell distribution width.

Between 2001 and 2019, 3,644 patients diagnosed with ICH were extracted from the MIMIC database. A total of 821 ICH patients were excluded, including 107 patients aged <18 years or ≥90 years, 568 patients admitted to the ICU for less than 24 h, and 146 patients with missing RDW or platelet information. Finally, 2,823 patients with ICH were included in the analysis (Figure 1). Table 1 demonstrates the characteristics of patients. The mean age of patients was 66.27 ± 15.02 years, 1,559 (55.22%) were male patients, 1,818 (64.40%) were White, 1,016 (35.99%) were admitted to ICU *via* emergency, 578 (20.47%) received vasopressor therapy, 646 (2.88%) received anticoagulation, 542 (19.20%) had sepsis, and 1,301 (46.09%) had neurodegeneration. The median length of ICU stay was 3.99 (2.08, 8.65) days, 96 (3.40%) patients died in the ICU, 2,446 (86.65%) patients were discharged from the ICU, and 281 (9.95%) patients experienced an ICU readmission. The median SOFA score was 3.00 (2.00, 5.00), the median GCS score was 13.00 (8.00, 13.30), and the median CCI score was 3.00 (1.00, 4.00). The median RPR value was 0.066 (0.053, 0.087). At the end of the 30-day follow-up, 799 (28.30%) patients died, and the median survival time was 30.00 (18.16, 30.00) days.

**Table 1:** Baseline characteristics of the patients with spontaneous intracerebral hemorrhage (ICH).

**Variables**	**Total (*n* = 2,823)**	**Survivors (*n* = 2,024)**	**Non-survivors (*n* = 799)**	***P***
Age, years, mean ± SD	66.27 ± 15.02	64.51 ± 15.09	70.72 ± 13.87	< 0.001
**Sex**, ***n*** **(%)**				0.014
Female	1264 (44.78)	877 (43.33)	387 (48.44)	
Male	1,559 (55.22)	1,147 (56.67)	412 (51.56)	
**Ethnicity**, ***n*** **(%)**				< 0.001
Black	258 (9.14)	195 (9.63)	63 (7.88)	
Others	375 (13.28)	286 (14.13)	89 (11.14)	
Unknown	372 (13.18)	225 (11.12)	147 (18.40)	
White	1,818 (64.40)	1,318 (65.12)	500 (62.58)	
**Insurance**, ***n*** **(%)**				< 0.001
Medicare	1,379 (48.85)	906 (44.76)	473 (59.20)	
Others	1,131 (40.06)	866 (42.79)	265 (33.17)	
Private	313 (11.09)	252 (12.45)	61 (7.63)	
**Admission type**, ***n*** **(%)**				0.005
Emergency	1,016 (35.99)	696 (34.39)	320 (40.05)	
Non-emergency	1,807 (64.01)	1,328 (65.61)	479 (59.95)	
**ICU type**, ***n*** **(%)**				< 0.001
MICU	338 (11.97)	224 (11.07)	114 (14.27)	
Others	316 (11.19)	264 (13.04)	52 (6.51)	
SICU	2,169 (76.83)	1,536 (75.89)	633 (79.22)	
**Ventilation**, ***n*** **(%)**				< 0.001
No	824 (29.19)	716 (35.38)	108 (13.52)	
Yes	1,999 (70.81)	1,308 (64.62)	691 (86.48)	
**Vasopressor**, ***n*** **(%)**				< 0.001
No	2,245 (79.53)	1,715 (84.73)	530 (66.33)	
Yes	578 (20.47)	309 (15.27)	269 (33.67)	
**Renal replacement therapy**, ***n*** **(%)**				< 0.001
No	2,747 (97.31)	1,987 (98.17)	760 (95.12)	
Yes	76 (2.69)	37 (1.83)	39 (4.88)	
**Congestive heart failure**, ***n*** **(%)**				< 0.001
No	2,441 (86.47)	1,780 (87.94)	661 (82.73)	
Yes	382 (13.53)	244 (12.06)	138 (17.27)	
**Sepsis**, ***n*** **(%)**				0.002
No	2,281 (80.80)	1,665 (82.26)	616 (77.10)	
Yes	542 (19.20)	359 (17.74)	183 (22.90)	
**Atrial fibrillation**, ***n*** **(%)**				< 0.001
No	2,235 (79.17)	1,640 (81.03)	595 (74.47)	
Yes	588 (20.83)	384 (18.97)	204 (25.53)	
**Hypertension**, ***n*** **(%)**				0.517
No	802 (28.41)	582 (28.75)	220 (27.53)	
Yes	2,021 (71.59)	1,442 (71.25)	579 (72.47)	
**Malignant cancer**, ***n*** **(%)**				0.854
No	2559 (90.65)	1836 (90.71)	723 (90.49)	
Yes	264 (9.35)	188 (9.29)	76 (9.51)	
**Diabetes**, ***n*** **(%)**				0.004
No	2,142 (75.88)	1,565 (77.32)	577 (72.22)	
Yes	681 (24.12)	459 (22.68)	222 (27.78)	
SBP, mmHg, Mean ± SD	139.91 ± 23.57	139.94 ± 22.64	139.82 ± 25.77	0.907
DBP, mmHg, Mean ± SD	73.77 ± 16.39	74.55 ± 16.14	71.82 ± 16.86	< 0.001
Respiratory rate, bpm, Mean ± SD	17.99 ± 4.33	17.87 ± 4.30	18.28 ± 4.39	0.025
Heart rate, bpm, Mean ± SD	81.61 ± 16.66	80.93 ± 16.10	83.33 ± 17.89	< 0.001
Temperature, °C, Mean ± SD	36.83 ± 0.73	36.84 ± 0.65	36.80 ± 0.92	0.347
SPO_2_, %, Mean ± SD	97.74 ± 3.44	97.64 ± 2.73	97.98 ± 4.77	0.063
SAPS II score, M (Q_1_, Q_3_)	33.00 (26.00, 41.00)	31.00 (24.00, 37.00)	40.00 (33.00, 50.00)	< 0.001
SOFA score, M (Q_1_, Q_3_)	3.00 (2.00, 5.00)	3.00 (2.00, 5.00)	5.00 (3.00, 7.00)	< 0.001
qSOFA score, M (Q_1_, Q_3_)	2.00 (1.00, 2.00)	2.00 (1.00, 2.00)	2.00 (1.00, 2.00)	< 0.001
GCS, M (Q_1_, Q_3_)	13.00 (8.00, 14.00)	13.00 (9.00, 14.00)	10.00 (5.00, 15.00)	< 0.001
CCI, M (Q_1_, Q_3_)	3.00 (1.00, 4.00)	3.00 (1.00, 4.00)	3.00 (2.00, 4.00)	< 0.001
WBC, K/uL, M (Q_1_, Q_3_)	10.40 (8.00, 13.30)	9.90 (7.75, 12.80)	11.80 (9.10, 14.70)	< 0.001
Hemoglobin, g/dL, Mean ± SD	12.05 ± 2.00	12.20 ± 1.94	11.68 ± 2.10	< 0.001
Hematocrit, %, Mean ± SD	35.83 ± 5.73	36.22 ± 5.54	34.84 ± 6.07	< 0.001
Creatinine, mg/dL, M (Q_1_, Q_3_)	0.90 (0.70, 1.10)	0.90 (0.70, 1.10)	0.90 (0.70, 1.30)	< 0.001
INR, ratio, M (Q_1_, Q_3_)	1.13 (1.10, 1.30)	1.11 (1.10, 1.20)	1.19 (1.10, 1.30)	< 0.001
Prothrombin time, sec, Mean ± SD	13.60 ± 4.17	13.41 ± 4.18	14.07 ± 4.09	< 0.001
Blood urea nitrogen, mg/dL, M (Q_1_, Q_3_)	16.00 (12.00, 22.00)	15.00 (12.00, 20.00)	18.00 (14.00, 26.00)	< 0.001
Glucose, mg/dL, M (Q_1_, Q_3_)	133.00 (111.00, 165.00)	128.00 (108.00, 155.00)	150.00 (123.00, 190.00)	< 0.001
Bicarbonate, mEq/L, Mean ± SD	23.85 ± 3.64	23.96 ± 3.44	23.57 ± 4.10	0.018
Sodium, mEq/L, Mean ± SD	139.11 ± 4.71	139.05 ± 4.42	139.27 ± 5.38	0.303
Potassium, mEq/L, Mean ± SD	3.94 ± 0.66	3.92 ± 0.63	3.98 ± 0.72	0.035
Chloride, mEq/L, Mean ± SD	103.70 ± 5.37	103.77 ± 5.13	103.52 ± 5.93	0.298
Urine output, mL, M (Q_1_, Q_3_)	1739.00 (1150.00, 2475.00)	1736.50 (1180.50, 2419.00)	1740.00 (1115.00, 2720.00)	0.653
**Mannitol**, ***n*** **(%)**				< 0.001
No	2,266 (80.27)	1,727 (85.33)	539 (67.46)	
Yes	557 (19.73)	297 (14.67)	260 (32.54)	
**Anticoagulation**, ***n*** **(%)**				< 0.001
No	2,177 (77.12)	1,515 (74.85)	662 (82.85)	
Yes	646 (22.88)	509 (25.15)	137 (17.15)	
**Blood transfusion**, ***n*** **(%)**				< 0.001
No	2,223 (78.75)	1,641 (81.08)	582 (72.84)	
Yes	600 (21.25)	383 (18.92)	217 (27.16)	
**Surgery**, ***n*** **(%)**				< 0.001
Craniotomy	81 (2.87)	73 (3.61)	8 (1.00)	
Minimally invasive surgery	251 (8.89)	170 (8.40)	81 (10.14)	
No surgery	2,491 (88.24)	1,781 (87.99)	710 (88.86)	
**Neurodegeneration**, ***n*** **(%)**				< 0.001
No	1,522 (53.91)	1,161 (57.36)	361 (45.18)	
Yes	1,301 (46.09)	863 (42.64)	438 (54.82)	
Length of hospital stay, days, M (Q_1_, Q_3_)	3.76 (2.01, 7.94)	3.84 (2.03, 8.68)	3.35 (1.98, 6.66)	< 0.001
ICU stay, days, M (Q1, Q3)	3.99 (2.08, 8.65)	4.14 (2.16, 9.59)	3.45 (1.95, 7.01)	< 0.001
**ICU status**, ***n*** **(%)**				< 0.001
ICU death	96 (3.40)	2 (0.10)	94 (11.76)	
ICU discharge	2,446 (86.65)	1,791 (88.49)	655 (81.98)	
ICU readmission	281 (9.95)	231 (11.41)	50 (6.26)	
Survival time within 30-day, days, M (Q_1_, Q_3_)	30.00 (18.16, 30.00)	30.00 (30.00, 30.00)	5.91 (2.94, 12.11)	< 0.001
RPR, M (Q_1_, Q_3_)	0.066 (0.053, 0.087)	0.065 (0.052, 0.083)	0.071 (0.056, 0.099)	< 0.001
**RPR**, ***n*** **(%)**				< 0.001
Tertile 1 (< 0.057)	941 (33.33)	717 (35.42)	224 (28.04)	
Tertile 2 (0.057–0.078)	941 (33.33)	699 (34.54)	242 (30.29)	
Tertile 3 (>0.078)	941 (33.33)	608 (30.04)	333 (41.68)	

ICU, intensive care unit; MICU, medical ICU; SICU, surgical ICU; SBP, systolic blood pressure; DBP, diastolic blood pressure; SPO_2_, saturation of peripheral oxygen; SAPS II, Simplified Acute Physiology Score; SOFA, Sequential Organ Failure Assessment; qSOFA, quick SOFA; GCS, Glasgow Coma Score; CCI, Charlson comorbidity index; WBC, white blood cell; INR, international normalized ratio; RPR, red cell distribution width-to-platelet ratio.

### Univariate Cox proportional hazard model

Table 2 shows the univariate analysis results of factors that may be associated with the risk of 30-day death in patients with ICH. Older age (HR = 1.03, 95%CI: 1.02–1.03), ventilation (HR = 3.08, 95%CI: 2.52–3.78), vasopressor (HR = 2.39, 95%CI: 2.06–2.77), renal replacement therapy (HR = 1.98, 95%CI: 1.44–2.74), sepsis (HR = 1.19, 95%CI: 1.01–1.40), atrial fibrillation (HR = 1.35, 95%CI: 1.15–1.58), higher respiratory rate (HR = 1.02, 95%CI: 1.01–1.03), higher heart rate (HR = 1.01, 95%CI: 1.01–1.01), higher SPO_2_ (HR = 1.04, 95%CI: 1.01–1.07), higher CCI (HR = 1.05, 95%CI: 1.02–1.08), higher WBC levels (HR = 1.01, 95%CI: 1.01–1.01), higher creatinine levels (HR = 1.06, 95%CI: 1.03–1.09), higher international normalized ratio (HR = 1.18, 95%CI: 1.07–1.31), higher prothrombin time (HR = 1.02, 95%CI: 1.01–1.03), higher blood urea nitrogen (HR = 1.01, 95%CI: 1.01–1.02), higher glucose (HR = 1.01, 95%CI: 1.01–1.01), higher potassium levels (HR = 1.11, 95%CI: 1.01–1.23), higher urine output (HR = 1.01, 95%CI: 1.01–1.01), mannitol (HR = 2.38, 95%CI: 2.05–2.76), blood transfusion (HR = 1.42, 95%CI: 1.22–1.66), surgery [minimally invasive surgery (HR = 3.72, 95%CI: 1.80–7.69) and no surgery (HR = 3.32, 95%CI: 1.65–6.66)], neurodegeneration (HR = 1.50, 95%CI: 1.31–1.73), and higher RPR value (HR = 4.04, 95%CI: 2.50–6.52) may be associated with higher risk of 30-day death. While being male (HR = 0.84, 95%CI: 0.73–0.96), private insurance (HR = 0.53, 95%CI: 0.41–0.69), admission to ICU *via* non-emergency (HR = 0.80, 95%CI: 0.70–0.93), higher DBP (HR = 0.99, 95%CI: 0.99–0.99), higher GCS (HR = 0.89, 95%CI: 0.88–0.91), higher hemoglobin levels (HR = 0.90, 95%CI: 0.87–0.93), higher hematocrit levels (HR = 0.97, 95%CI: 0.96–0.98), higher bicarbonate levels (HR = 0.97, 95%CI: 0.95–0.99), anticoagulation (HR = 0.62, 95%CI: 0.52–0.75), and longer length of hospital stay (HR = 0.95, 95%CI: 0.94–0.97) may be associated with a decreased risk of 30-day death.

**Table 2:** Univariate Cox proportional hazard model of factors associated with the risk of 30-day death in patients with spontaneous intracerebral hemorrhage (ICH).

**Variables**	**β**	**S.E**	**χ^2^**	**HR (95%CI)**	***P***
Age	0.025	0.003	88.720	1.03 (1.02–1.03)	< 0.001
**Sex**					
Female				Ref	
Male	−0.176	0.071	6.160	0.84 (0.73–0.96)	0.013
**Race**					
Black				Ref	
Others	0.027	0.165	0.027	1.03 (0.74–1.42)	0.869
Unknown	0.587	0.151	15.194	1.80 (1.34–2.42)	< 0.001
White	0.142	0.134	1.132	1.15 (0.89–1.50)	0.287
**Insurance**					
Medicare				Ref	
Others	−0.425	0.077	30.633	0.65 (0.56–0.76)	< 0.001
Private	−0.632	0.136	21.554	0.53 (0.41–0.69)	< 0.001
**Admission type**					
Emergency				Ref	
Non-emergency	−0.218	0.072	9.086	0.80 (0.70–0.93)	0.003
**ICU type**					
MICU				Ref	
Others	−0.825	0.167	24.304	0.44 (0.32–0.61)	< 0.001
SICU	−0.161	0.102	2.511	0.85 (0.70–1.04)	0.113
**Ventilation**					
No				Ref	
Yes	1.126	0.104	118.270	3.08 (2.52–3.78)	< 0.001
**Vasopressor**					
No				Ref	
Yes	0.872	0.075	135.379	2.39 (2.06–2.77)	< 0.001
**Renal replacement therapy**					
No				Ref	
Yes	0.684	0.164	17.377	1.98 (1.44–2.74)	< 0.001
**Sepsis**					
No				Ref	
Yes	0.171	0.084	4.128	1.19 (1.01–1.40)	0.042
**Atrial fibrillation**					
No				Ref	
Yes	0.298	0.081	13.484	1.35 (1.15–1.58)	< 0.001
SBP	−0.000	0.002	0.027	1.00 (1.00–1.00)	0.864
DBP	−0.009	0.002	17.242	0.99 (0.99–0.99)	< 0.001
Respiratory rate	0.018	0.008	4.805	1.02 (1.01–1.03)	0.029
Heart rate	0.007	0.002	11.104	1.01 (1.01–1.01)	< 0.001
Temperature	−0.068	0.052	1.699	0.93 (0.84–1.03)	0.192
SPO_2_	0.043	0.014	8.895	1.04 (1.01–1.07)	0.003
GCS	−0.112	0.009	155.705	0.89 (0.88–0.91)	< 0.001
CCI	0.046	0.015	9.812	1.05 (1.02–1.08)	0.002
WBC	0.007	0.001	22.206	1.01 (1.01–1.01)	< 0.001
Hemoglobin	−0.102	0.017	34.609	0.90 (0.87–0.93)	< 0.001
Hematocrit	−0.034	0.006	30.587	0.97 (0.96–0.98)	< 0.001
Creatinine	0.054	0.014	14.609	1.06 (1.03–1.09)	< 0.001
INR	0.169	0.051	11.127	1.18 (1.07–1.31)	< 0.001
Prothrombin time	0.018	0.005	13.437	1.02 (1.01–1.03)	< 0.001
Blood urea nitrogen	0.012	0.001	72.127	1.01 (1.01–1.02)	< 0.001
Glucose	0.005	0.000	170.186	1.01 (1.01–1.01)	< 0.001
Bicarbonate	−0.027	0.010	7.474	0.97 (0.95–0.99)	0.006
Sodium	0.012	0.008	2.172	1.01 (1.00–1.03)	0.141
Potassium	0.108	0.051	4.508	1.11 (1.01–1.23)	0.034
Chloride	−0.007	0.007	1.066	0.99 (0.98–1.01)	0.303
Urine output	0.000	0.000	26.646	1.01 (1.01–1.01)	< 0.001
**Mannitol**					
No				Ref	
Yes	0.868	0.076	131.939	2.38 (2.05–2.76)	< 0.001
**Anticoagulation**					
No				Ref	
Yes	−0.472	0.094	25.328	0.62 (0.52–0.75)	< 0.001
**Blood transfusion**					
No				Ref	
Yes	0.351	0.080	19.518	1.42 (1.22–1.66)	< 0.001
**Surgery**					
Craniotomy				Ref	
Minimally invasive surgery	1.313	0.371	12.554	3.72 (1.80–7.69)	< 0.001
No surgery	1.199	0.356	11.376	3.32 (1.65–6.66)	< 0.001
**Neurodegeneration**					
No				Ref	
Yes	0.406	0.071	32.649	1.50 (1.31–1.73)	< 0.001
**RPR**					
Tertile 1 (< 0.057)				Ref	
Tertile 2 (0.057-0.078)	0.077	0.093	0.690	1.08 (0.90–1.30)	0.406
Tertile 3 (>0.078)	0.457	0.086	27.929	1.58 (1.33–1.87)	< 0.001

S.E, standard error; HR, hazard ratio; 95%CI, confidence interval; Ref, reference; ICU, intensive care unit; MICU, medical ICU; SICU, surgical ICU; SBP, systolic blood pressure; DBP, diastolic blood pressure; SPO2, saturation of peripheral oxygen; GCS, Glasgow Coma Score; CCI, Charlson comorbidity index; WBC, white blood cell; INR, international normalized ratio; RPR, red cell distribution width to platelet ratio.

### Association between RPR and the risk of 30-day death in patients with ICH

The results of univariate and multivariate Cox proportional hazard models for the relationship between RPR and the risk of 30-day death in patients with ICH are demonstrated in Table 3. Compared with tertile 1, the tertile 3 of RPR levels (HR = 1.58, 95%CI: 1.33–1.87) were associated with an increased risk of 30-day death in patients with ICH. After adjusting for age, ethnicity, ICU type, ventilation, vasopressor, renal replacement therapy, sepsis, heart rate, SPO_2_, hemoglobin, blood urea nitrogen, glucose, urine output, mannitol, anticoagulation, surgery, and neurodegeneration, the tertile 3 of RPR levels (HR = 1.37, 95%CI: 1.15–1.64) were still associated with a higher risk of 30-day death.

**Table 3:** Association between RPR and the risk of 30-day death in patients with ICH.

**Variables**	**Univariate**		**Multivariate**	
	**HR (95%CI)**	***P***	**HR (95%CI)**	***P***
**RPR**				
Tertile 1 (< 0.057)	Ref		Ref	
Tertile 2 (0.057–0.078)	1.08 (0.90–1.30)	0.406	1.09 (0.91–1.32)	0.334
Tertile 3 (>0.078)	1.58 (1.33–1.87)	< 0.001	1.37 (1.15–1.64)	< 0.001

RPR, red cell distribution width-to-platelet ratio; ICH, spontaneous intracerebral hemorrhage; HR, hazard ratio; 95%CI, confidence interval.

Multivariate Cox proportional hazard model adjusted for age, ethnicity, ICU type, ventilation, vasopressor, renal replacement therapy, sepsis, heart rate, SPO_2_, hemoglobin, blood urea nitrogen, glucose, urine output, mannitol, anticoagulation, surgery, and neurodegeneration.

Furthermore, 281 (9.95%) patients experienced an ICU readmission. Factors associated with ICU readmission in patients with ICH were analyzed (Supplementary Table 2). The results showed that the tertile 1 (HR = 1.45, 95%CI: 1.07–1.97) and tertile 3 (HR = 1.56, 95%CI: 1.13–2.15) of RPR levels were associated with a higher risk of ICU readmissions in patients with ICH compared with tertile 2 of RPR levels.

**Figure 2:**
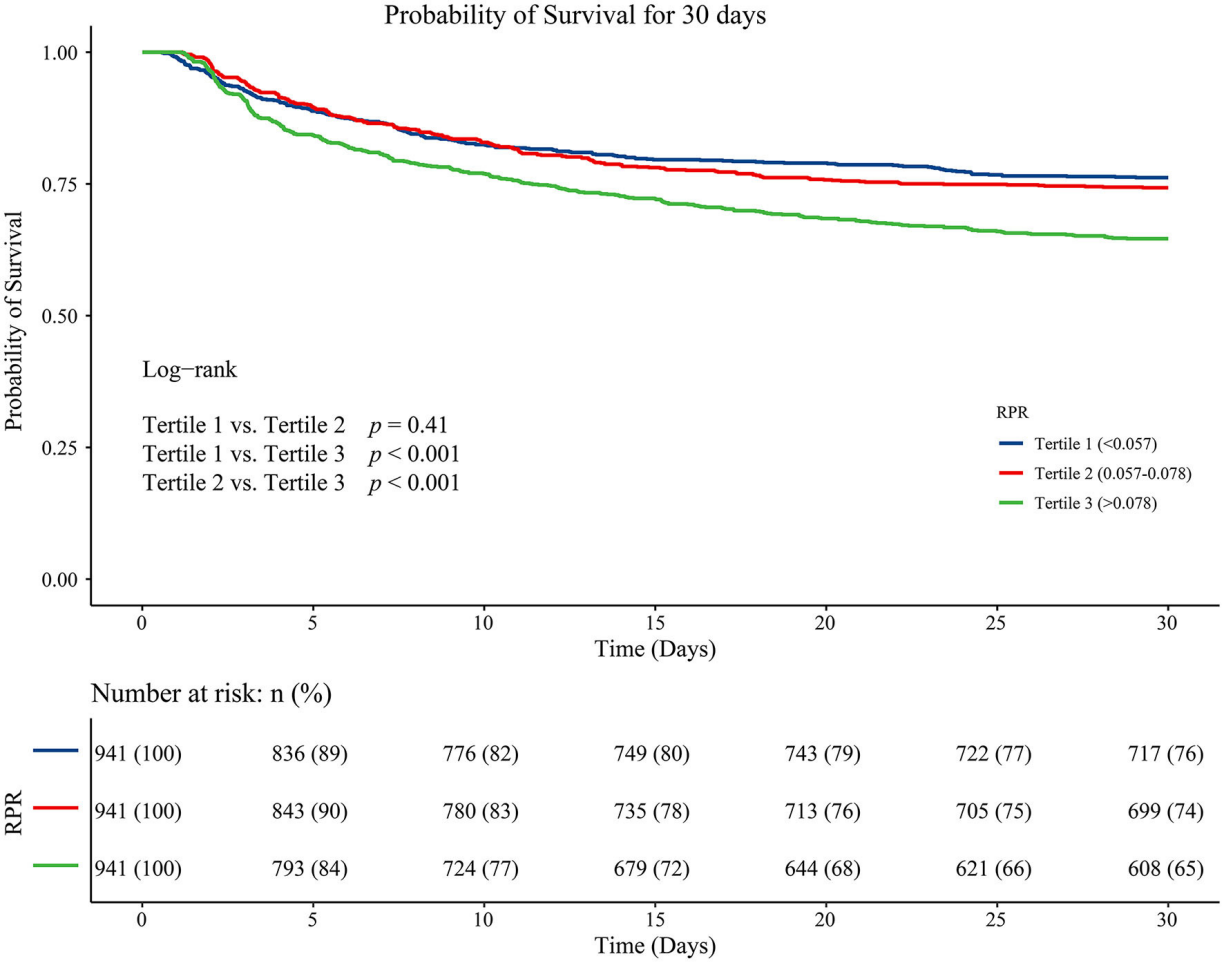
Kaplan–Meier survival curves for 30-day death in ICH patients with different RPR levels. ICH, spontaneous intracerebral hemorrhage; RPR, red cell distribution width to platelet ratio.

The K-M survival curves showed that patients with tertile 3 of RPR levels had a higher risk of 30-day death compared with tertile 1 and tertile 2 of RPR (*P* < 0.001) (Figure 2). Table 4 presents the comparison of different tools for the identification of 30-day death in patients with ICH. The C-index was 0.772 (95%CI: 0.757–0.786) for the RPR tool, 0.656 (95%CI: 0.636–0.675) for the SOFA tool, 0.712 (95%CI: 0.695–0.729) for the SAPS II tool, and 0.529 (95%CI: 0.511–0.548) for the qSOFA tool, and the C-index of the RPR was higher than that of the other tools (all *P* < 0.001).

**Table 4:** C-index of different tools for the identification of 30-day death in patients with spontaneous intracerebral hemorrhage (ICH).

**Tools**	**C-index (95%CI)**	**Statistics**	***P***
RPR	0.772 (0.757–0.786)	Ref	
SOFA	0.656 (0.636–0.675)	95.60	< 0.001
SAPS II	0.712 (0.695–0.729)	36.11	< 0.001
qSOFA	0.529 (0.511–0.548)	399.11	< 0.001

RPR, red cell distribution width to platelet ratio; SOFA, Sequential Organ Failure Assessment; SAPS II, Simplified Acute Physiology Score; qSOFA, quick SOFA.

### Influence of RPR on 30-day death in ICH patients based on different populations

The association between RPR and the risk of 30-day death in ICH patients was stratified according to age (<65 and ≥65 years), sex (female and male), GCS score (<14 and ≥14), CCI score (<4 and ≥4), and sepsis (yes and no) (Table 5). In patients of different ages, the tertile 3 of RPR levels were found to be associated with a higher risk of 30-day death in patients aged <65 years (HR = 1.77, 95%CI: 1.29–2.43) and ≥65 years (HR = 1.30, 95%CI: 1.05–1.61). The tertile 2 (HR = 1.32, 95%CI: 1.03–1.71) and tertile 3 (HR = 1.75, 95%CI: 1.35–2.26) of RPR levels were associated with an increased risk of 30-day death in female patients but not in male patients (*P*>0.157). Among patients with different GCS scores, the tertile 3 of RPR levels was correlated with a higher risk of 30-day death in patients with GCS score <14 (HR = 1.65, 95%CI: 1.27–2.14) but not in patients with GCS score ≥14 (*P* = 0.058). In addition, the tertile 3 of RPR levels were related to a higher risk of 30-day death in patients with CCI ≥4 (HR = 1.45, 95%CI: 1.17–1.80), whereas no association was found in patients with CCI <4 (*P* = 0.188). For sepsis, the tertile 3 of RPR levels were associated with a higher risk of 30-day death in patients with (HR = 1.66, 95%CI: 1.13–2.43) and without (HR = 1.32, 95%CI: 1.08–1.61) sepsis.

**Table 5:** Association of RPR with the risk of 30-day death in ICH patients stratified by age, sex, GCS score, and CCI score.

**Subgroups**	**Univariate**		**Multivariate**	
	**HR (95%CI)**	***P***	**HR (95%CI)**	***P***
**Age**<**65 (*****n*** = **1,211)**				
Tertile 1 of RPR (< 0.057)	Ref		Ref	
Tertile 2 of RPR (0.057–0.078)	0.90 (0.65–1.26)	0.555	1.07 (0.76–1.50)	0.699
Tertile 3 of RPR (>0.078)	1.99 (1.49–2.65)	< 0.001	1.77 (1.29-2.43)	< 0.001
**Age** ≥**65 (*****n*** = **1,612)**				
Tertile 1 of RPR	Ref		Ref	
Tertile 2 of RPR	1.06 (0.85-1.32)	0.596	1.13 (0.91-1.41)	0.272
Tertile 3 of RPR	1.26 (1.03-1.56)	0.028	1.30 (1.05-1.61)	0.016
**Sex-female (*****n*** = **1,264)**				
Tertile 1 of RPR	Ref		Ref	
Tertile 2 of RPR	1.36 (1.06–1.74)	0.016	1.32 (1.03–1.71)	0.029
Tertile 3 of RPR	1.74 (1.36–2.22)	< 0.001	1.75 (1.35–2.26)	< 0.001
**Sex-male (*****n*** = **1,559)**				
Tertile 1 of RPR	Ref		Ref	
Tertile 2 of RPR	0.89 (0.68–1.16)	0.372	0.90 (0.69–1.19)	0.473
Tertile 3 of RPR	1.50 (1.18–1.90)	< 0.001	1.21 (0.94–1.56)	0.139
**GCS**<**14 (*****n*** = **1,220)**				
Tertile 1 of RPR	Ref		Ref	
Tertile 2 of RPR	1.10 (0.83–1.45)	0.495	1.22 (0.92–1.61)	0.160
Tertile 3 of RPR	1.53 (1.19–1.97)	0.001	1.65 (1.27–2.14)	< 0.001
**GCS** ≥**14 (*****n*** = **1,603)**				
Tertile 1 of RPR	Ref		Ref	
Tertile 2 of RPR	1.04 (0.82–1.32)	0.756	1.02 (0.80–1.31)	0.873
Tertile 3 of RPR	1.47 (1.17–1.85)	< 0.001	1.26 (0.99–1.60)	0.058
**CCI**<**4 (*****n*** = **1,175)**				
Tertile 1 of RPR	Ref		Ref	
Tertile 2 of RPR	1.06 (0.81–1.39)	0.679	0.91 (0.66–1.25)	0.574
Tertile 3 of RPR	1.42 (1.08–1.87)	0.011	1.24 (0.90–1.71)	0.188
**CCI** ≥**4 (*****n*** = **1,648)**				
Tertile 1 of RPR	Ref		Ref	
Tertile 2 of RPR	1.09 (0.86–1.40)	0.469	1.19 (0.95–1.50)	0.131
Tertile 3 of RPR	1.66 (1.33–2.08)	< 0.001	1.45 (1.17–1.80)	< 0.001
**Sepsis-No (*****n*** = **2,281)**				
Tertile 1 of RPR	Ref		Ref	
Tertile 2 of RPR	1.08 (0.88–1.32)	0.462	1.08 (0.88–1.32)	0.486
Tertile 3 of RPR	1.49 (1.23–1.80)	< .001	1.32 (1.08–1.61)	0.006
**Sepsis-Yes (*****n*** = **542)**				
Tertile 1 of RPR	Ref		Ref	
Tertile 2 of RPR	1.07 (0.71–1.60)	0.761	1.03 (0.68–1.57)	0.888
Tertile 3 of RPR	1.91 (1.33–2.75)	< 0.001	1.66 (1.13–2.43)	0.010

RPR, red cell distribution width-to-platelet ratio; ICH, spontaneous intracerebral hemorrhage; HR, hazard ratio; 95%CI, confidence interval, GCS, Glasgow Coma Score; CCI, Charlson comorbidity index.

Multivariate Cox proportional hazard model adjusted for age, ethnicity, ICU type, ventilation, vasopressor, renal replacement therapy, heart rate, SPO_2_, hemoglobin, blood urea nitrogen, glucose, urine output, mannitol, anticoagulation, surgery, and neurodegeneration.

### Predictive ability of RPR for 30-day death in patients with ICH

**Figure 3:**
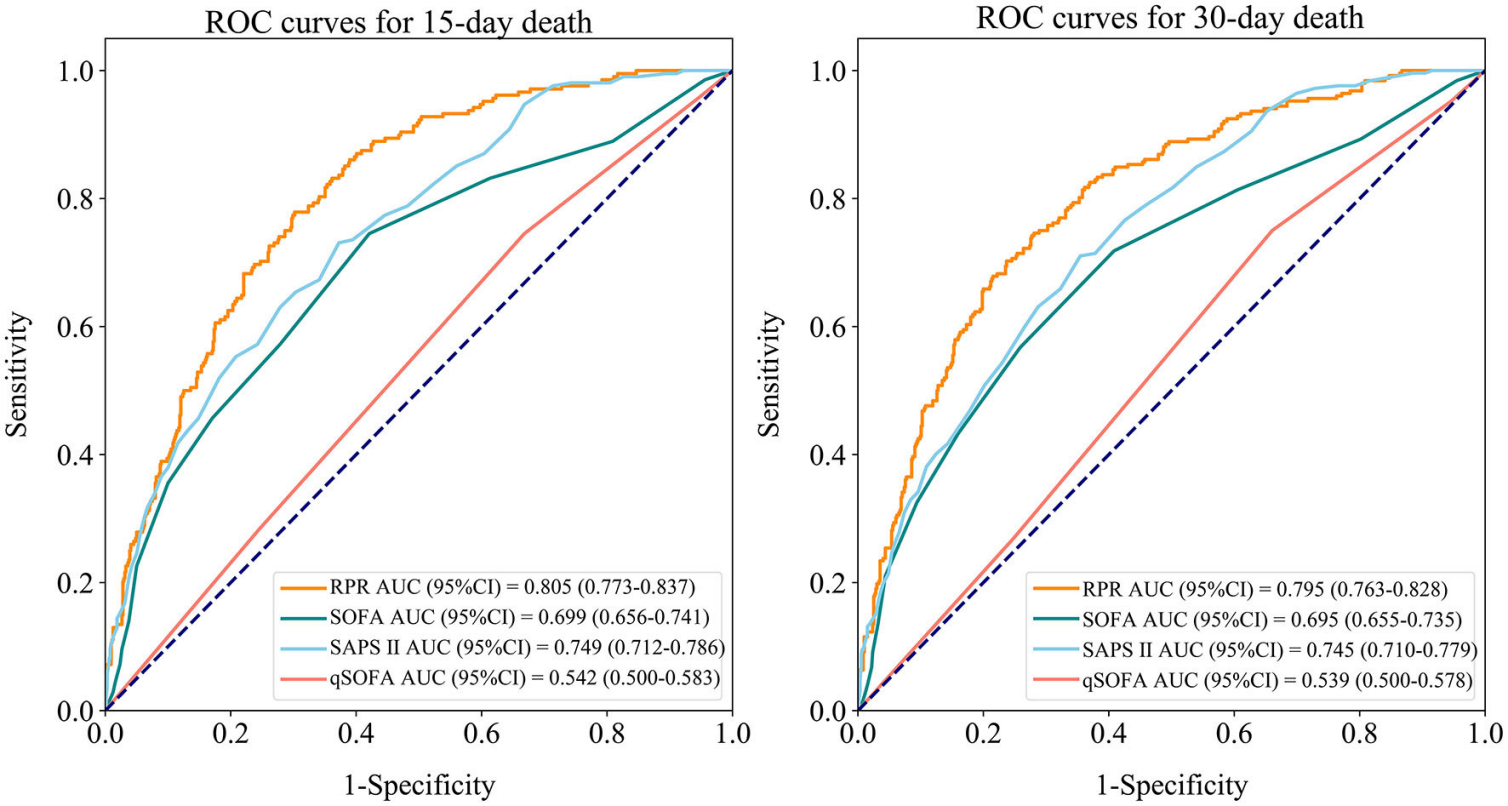
Receiver operating characteristic (ROC) curves for RPR, SOFA, SAPS II, and qSOFA to predict 30-day and 15-day death in ICH patients (testing set). RPR, red cell distribution width to platelet ratio; SOFA, Sequential Organ Failure Assessment; SAPS II, Simplified Acute Physiology Score; qSOFA, quick SOFA; ICH, spontaneous intracerebral hemorrhage.

**Figure 4:**
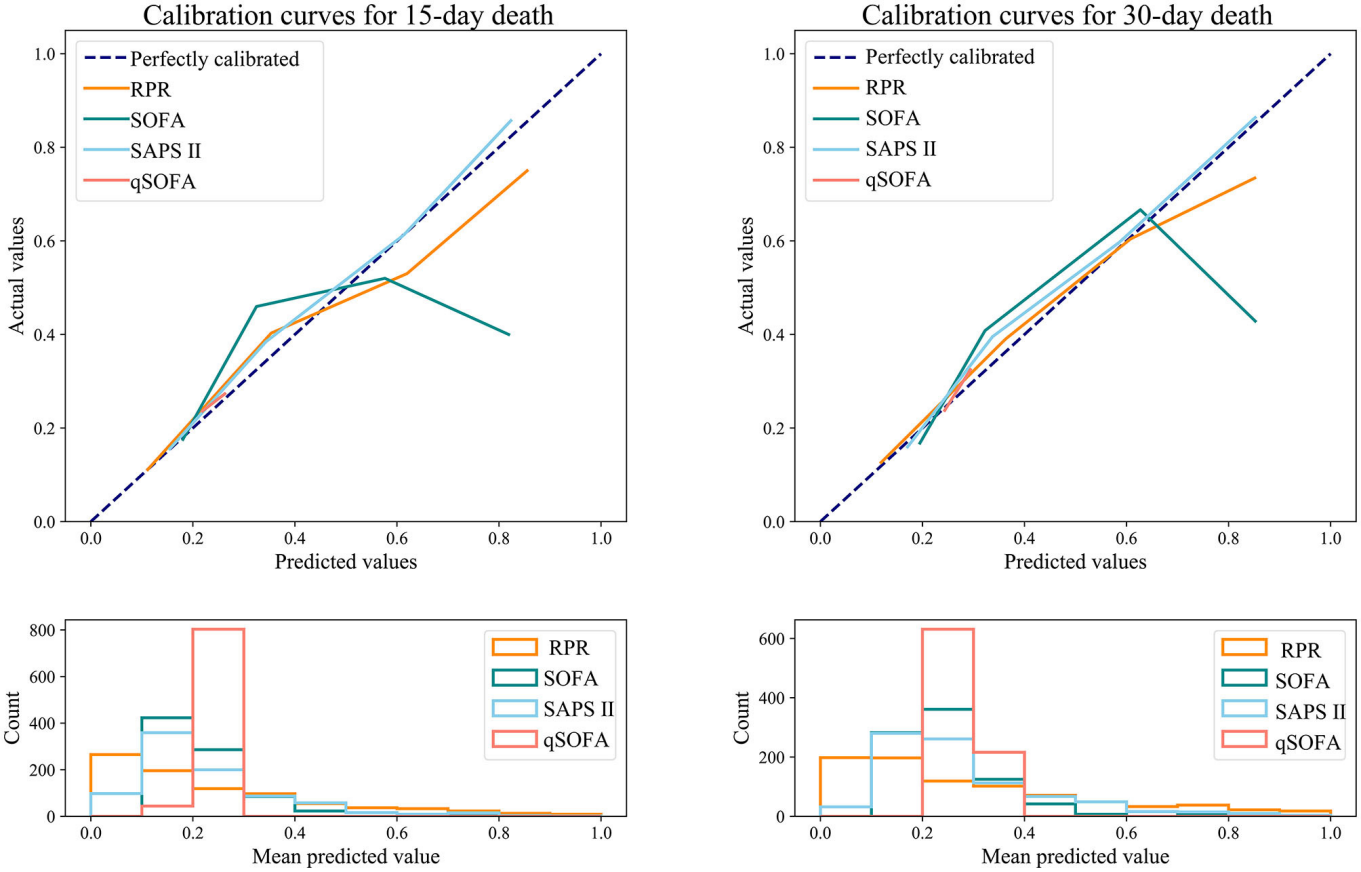
Calibration curves for RPR, SOFA, SAPS II, and qSOFA to predict 30-day and 15-day death in ICH patients (testing set). RPR, red cell distribution width to platelet ratio; SOFA, Sequential Organ Failure Assessment; SAPS II, Simplified Acute Physiology Score; qSOFA, quick SOFA; ICH, spontaneous intracerebral hemorrhage.

Table 6 shows the predictive ability of RPR, SOFA, SAPS II, and qSOFA for 30-day and 15-day death in patients with ICH, and Figure 3 demonstrates the ROC curves for these tools. The AUCs of the RPR, SOFA, SAPS II, and qSOFA tools for predicting 30-day death in patients with ICH in the testing set were 0.795 (95%CI: 0.763–0.828), 0.695 (95%CI: 0.655–0.735), 0.745 (95%CI: 0.710–0.779), and 0.539 (95%CI: 0.500–0.578), respectively. The RPR tool had the highest AUC for predicting 30-day death in patients with ICH compared with other tools (all *P* < 0.001). The RPR tool also had the highest AUC for predicting 15-day death in patients with ICH, with an AUC of 0.805 (95%CI: 0.773–0.837). The calibration curves showed no deviation between the predicted and observed probability of RPR in predicting 30-day and 15-day death in patients with ICH (Figure 4).

**Table 6:** Predictive ability of RPR, SOFA, SAPS II, and qSOFA for 30-day and 15-day death in patients with ICH.

**Outcomes**	**Dataset**	**Tools**	**AUC (95%CI)**	**Statistics**	***P***
30-day death	Training set	RPR	0.805 (0.785–0.826)	Ref	
		SOFA	0.679 (0.651–0.707)	7.52623	< 0.001
		SAPS II	0.741 (0.717–0.765)	4.60361	< 0.001
		qSOFA	0.539 (0.513–0.565)	16.28586	< 0.001
	Testing set	RPR	0.795 (0.763–0.828)	Ref	
		SOFA	0.695 (0.655v0.735)	4.28337	< 0.001
		SAPS II	0.745 (0.710–0.779)	2.54870	0.011
		qSOFA	0.539 (0.500–0.578)	10.34407	< 0.001
15-day death	Training set	RPR	0.811 (0.790–0.832)	Ref	
		SOFA	0.661 (0.630–0.691)	8.30466	< 0.001
		SAPS II	0.739 (0.713–0.765)	4.84972	< 0.001
		qSOFA	0.525 (0.497–0.553)	16.39272	< 0.001
	Testing set	RPR	0.805 (0.773–0.837)	Ref	
		SOFA	0.699 (0.656–0.741)	4.16820	< 0.001
		SAPS II	0.749 (0.712–0.786)	2.59159	0.01
		qSOFA	0.542 (0.500–0.583)	9.93301	< 0.001

ICH, spontaneous intracerebral hemorrhage; RPR, red cell distribution width to platelet ratio; SOFA, Sequential Organ Failure Assessment; SAPS II, Simplified Acute Physiology Score; qSOFA, quick SOFA; AUC, area under receiver-operating characteristic curve.

## Discussion

The present study investigated the association between RPR and 30-day death in patients with ICH and explored the predictive value of RPR for 30-day death. We found elevated RPR levels were correlated with an increased risk of 30-day death in patients with ICH. In addition, RPR had a good predictive ability for 30-day death in patients with ICH, with an AUC value of 0.795.

Inflammatory response plays an important role in the pathophysiological processes of brain injury after ICH (16, 17). Both RDW and RPR have been reported to be associated with mortality in patients with ICH (15, 18). Pinho et al. found that elevated RDW levels were independently associated with increased 30-day mortality in patients with ICH (18). Lehmann et al. showed that ICH patients with elevated RPR in the admission laboratory were more likely to die within 90 days of bleeding (15). In addition, RPR was considered a strong predictor of prognosis for a variety of diseases such as breast cancer (19), glioblastoma (20), myocardial infarction (21), and acute traumatic brain injury (14). The use of RPR for prognosis assessment of disease is related to the physiological function of RDW and platelets. RDW represents heterogeneity in erythrocyte size, with higher values indicating greater variability. Abnormally elevated RDW is associated with oxidative stress, chronic inflammatory responses, and impaired erythropoiesis (22, 23). Inflammatory cytokines can affect the survival of circulating erythrocytes, reduce deformation, inhibit maturation, and lead to an increase in RDW (24, 25). Excessive oxidative stress reduces the activity of red blood cells (23). Moreover, elevated RDW levels were found to be associated with the risk of recurrence of small artery occlusion (26). Pathological changes of cerebral small vessels can cause small artery occlusion and also involve deep ICH. Platelets are also a common laboratory indicator, and thrombocytopenia may increase the risk of bleeding (27). The most common cause of thrombocytopenia in critically ill patients is severe infection and/or inflammation, which causes circulating thrombocytopenia primarily through abnormal platelet-vessel wall interactions and abnormal platelet activation (28). The present study found that elevated RPR levels were related to an increased risk of 30-day death in patients with ICH, which was consistent with the previous study (15). Compared with previous studies, our study provided stronger evidence based on a large sample of the database. Second, we not only analyzed the association between RPR and death risk in ICH patients but also further analyzed the predictive value of RPR for death in ICH patients. Furthermore, the outcome of our study was 30-day death in patients with ICH compared to 90-day death in the previous study, which may be a difference. Pinho et al. (18) showed that the prognostic value of the same indicator for patients with ICH may vary depending on the length of time to assess death (30-day death or more).

Our results showed that compared with SOFA, SAPS II, and qSOFA scores, RPR had the highest AUC for predicting 30-day death in ICH patients, with an AUC value of 0.806. The calibration curves demonstrated that no deviation was observed between the predicted and observed probability of RPR in predicting 30-day death. These results suggested that RPR had a good predictive ability for 30-day death in patients with ICH. RPR as an independent marker of inflammation may be used in the prognostic assessment of patients with ICH. In the subgroup analysis between RPR and 30-day death risk, elevated RPR levels were observed to be linked to an increased 30-day death risk in patients aged <65, aged ≥65 years, GCS score <9, GCS score ≥9, female patients, and CCI ≥3, whereas no association was observed in male patients. The association between RPR and 30-day death in patients with ICH varies by gender, which may require further study. Several studies have reported sex differences in outcomes for patients with ICH (29–31). However, these studies did not reach consistent results and did not provide a reasonable explanation for the sex differences. Umeano et al. suggested that there may be an interaction between sex and age that influences the outcomes of patients with ICH (29), but prospective studies are needed to investigate this hypothesis.

Our study provided evidence of the relationship between RPR and 30-day death in ICH patients based on a large sample of data from the MIMIC database. The association between RPR and 30-day death was then analyzed stratified by age, sex, GCS score, CCI score, and sepsis. In addition, we investigated the predictive value of RPR levels for 30-day death in patients with ICH and compared it with other tools such as SOFA, SAPS II, and qSOFA. However, several limitations should be taken into account when interpreting our results. First, this study was based on single-center data from the MIMIC database, and further studies may require prospective multicenter studies to provide more evidence. Second, retrospective study design is subject to reporting bias and thus has an impact on the results, and prospective studies are needed. Third, the MIMIC database lacks imaging-related records to capture indicators affecting the prognosis of ICH such as hematoma volume, but we used the GCS score to reflect the organic status and severity of patients with ICH, and the corresponding subgroup analysis was performed. Fourth, both neutrophil-to-lymphocyte ratio (NLR) and neutrophil-to-platelet ratio (NPR) have been reported to be correlated with ICH. However, it was not possible to compare the effects of RPR, NLR, and NPR because of the high rate of missing data (>80%) for neutrophils and lymphocytes in this study population. Fifth, we were unable to analyze the relationship between RPR and different subtypes of ICH (e.g., deep ICH and lobar ICH) due to the limitations of the MIMIC database. Sixth, the present study focused on the association of RPR levels at patient admission with death, while the association between dynamic changes in RPR levels during treatment (e.g., RPR trajectory) and death may merit further study. Seventh, the predictive value of RPR in combination with other biomarkers such as S100 calcium-binding protein B and thioredoxin for death in patients with ICH needs to be further explored. Eighth, future studies could explore the association between RPR and disease processes such as hematoma expansion and neurological deterioration in patients with ICH, which could contribute to the understanding of the disease process of ICH.

## Conclusion

The current study indicated that elevated RPR levels were associated with a higher 30-day death risk in patients with ICH. RPR levels showed good predictive ability for 30-day death in patients with ICH compared with other tools. Increased RPR levels may provide clinicians with a signal of an elevated risk of death in patients with ICH.

## Data availability statement

The data analyzed in this study was obtained from the Medical Information Mart for Intensive Care (MIMIC)-III and MIMIC-IV databases, the following licenses/restrictions apply: To access the files, users must be credentialed users, complete the required training (CITI Data or Specimens Only Research) and sign the data use agreement for the project. Requests to access these datasets should be directed to PhysioNet, https://physionet.org/, https://doi.org/10.13026/C2XW26, and https://doi.org/10.13026/6mm1-ek67.

## Ethics statement

Ethical review and approval was not required for the study on human participants in accordance with the local legislation and institutional requirements. Written informed consent from the patients/participants or patients/participants' legal guardian/next of kin was not required to participate in this study in accordance with the national legislation and the institutional requirements.

## Author contributions

HL designed the study and wrote the manuscript. PL, LG, JF, CY, DZ, and LC collected, analyzed, and interpreted the data. HL critically reviewed, edited, and approved the manuscript. All authors read and approved the final manuscript.
