# Supplementary material for: Predictive value of admission red cell distribution width-to-platelet ratio for 30-day death in patients with spontaneous intracerebral hemorrhage: an analysis of the MIMIC database
Source: Front Neurol. 2023 Oct 18;14:1221335. doi: 10.3389/fneur.2023.1221335 (PMC10618669; doi:10.3389/fneur.2023.1221335)
Supplement: Supplementary file 1 [file Table_1.DOCX]

**Supplement Table 1**. Characteristics of patients with ICH in the training set and testing set.

| Variables | Total (n=2823) | Testing set (n=847) | Training set (n=1976) | Statistics | *P* |
| --- | --- | --- | --- | --- | --- |
| Age, years, Mean ± SD | 66.27 ± 15.02 | 66.00 ± 15.19 | 66.39 ± 14.95 | t=-0.63 | 0.528 |
| Sex, n (%) |  |  |  | χ^2^=0.021 | 0.885 |
| Female | 1264 (44.78) | 381 (44.98) | 883 (44.69) |  |  |
| Male | 1559 (55.22) | 466 (55.02) | 1093 (55.31) |  |  |
| Ethnicity, n (%) |  |  |  | χ^2^=1.122 | 0.772 |
| Black | 258 (9.14) | 72 (8.50) | 186 (9.41) |  |  |
| Others | 375 (13.28) | 118 (13.93) | 257 (13.01) |  |  |
| Unknown | 372 (13.18) | 108 (12.75) | 264 (13.36) |  |  |
| White | 1818 (64.40) | 549 (64.82) | 1269 (64.22) |  |  |
| Insurance, n (%) |  |  |  | χ^2^=1.070 | 0.586 |
| Medicare | 1379 (48.85) | 424 (50.06) | 955 (48.33) |  |  |
| Others | 1131 (40.06) | 327 (38.61) | 804 (40.69) |  |  |
| Private | 313 (11.09) | 96 (11.33) | 217 (10.98) |  |  |
| Admission type, n (%) |  |  |  | χ^2^=0.059 | 0.808 |
| Emergency | 1016 (35.99) | 302 (35.66) | 714 (36.13) |  |  |
| Non-emergency | 1807 (64.01) | 545 (64.34) | 1262 (63.87) |  |  |
| ICU type, n (%) |  |  |  | χ^2^=0.765 | 0.682 |
| MICU | 338 (11.97) | 108 (12.75) | 230 (11.64) |  |  |
| Others | 316 (11.19) | 96 (11.33) | 220 (11.13) |  |  |
| SICU | 2169 (76.83) | 643 (75.91) | 1526 (77.23) |  |  |
| Ventilation, n (%) |  |  |  | χ^2^=0.223 | 0.637 |
| No | 824 (29.19) | 242 (28.57) | 582 (29.45) |  |  |
| Yes | 1999 (70.81) | 605 (71.43) | 1394 (70.55) |  |  |
| Vasopressor, n (%) |  |  |  | χ^2^=0.133 | 0.716 |
| No | 2245 (79.53) | 670 (79.10) | 1575 (79.71) |  |  |
| Yes | 578 (20.47) | 177 (20.90) | 401 (20.29) |  |  |
| Renal replacement therapy, n (%) |  |  |  | χ^2^=3.920 | 0.048 |
| No | 2747 (97.31) | 832 (98.23) | 1915 (96.91) |  |  |
| Yes | 76 (2.69) | 15 (1.77) | 61 (3.09) |  |  |
| Congestive heart failure, n (%) |  |  |  | χ^2^=1.270 | 0.260 |
| No | 2441 (86.47) | 723 (85.36) | 1718 (86.94) |  |  |
| Yes | 382 (13.53) | 124 (14.64) | 258 (13.06) |  |  |
| Sepsis, n (%) |  |  |  | χ^2^=0.002 | 0.968 |
| No | 2281 (80.80) | 684 (80.76) | 1597 (80.82) |  |  |
| Yes | 542 (19.20) | 163 (19.24) | 379 (19.18) |  |  |
| Atrial fibrillation, n (%) |  |  |  | χ^2^=1.111 | 0.292 |
| No | 2235 (79.17) | 681 (80.40) | 1554 (78.64) |  |  |
| Yes | 588 (20.83) | 166 (19.60) | 422 (21.36) |  |  |
| Hypertension, n (%) |  |  |  | χ^2^=0.094 | 0.759 |
| No | 802 (28.41) | 244 (28.81) | 558 (28.24) |  |  |
| Yes | 2021 (71.59) | 603 (71.19) | 1418 (71.76) |  |  |
| Malignant cancer, n (%) |  |  |  | χ^2^=0.286 | 0.593 |
| No | 2559 (90.65) | 764 (90.20) | 1795 (90.84) |  |  |
| Yes | 264 (9.35) | 83 (9.80) | 181 (9.16) |  |  |
| Diabetes, n (%) |  |  |  | χ^2^=2.264 | 0.132 |
| No | 2142 (75.88) | 627 (74.03) | 1515 (76.67) |  |  |
| Yes | 681 (24.12) | 220 (25.97) | 461 (23.33) |  |  |
| SBP, mmHg, Mean ± SD | 139.97 ± 23.56 | 139.71 ± 23.29 | 140.07 ± 23.68 | t=-0.37 | 0.708 |
| DBP, mmHg, Mean ± SD | 73.77 ± 16.39 | 73.35 ± 16.46 | 73.95 ± 16.36 | t=-0.89 | 0.373 |
| Respiratory rate, bpm, Mean ± SD | 17.99 ± 4.33 | 18.10 ± 4.22 | 17.94 ± 4.37 | t=0.94 | 0.347 |
| Heart rate, bpm, Mean ± SD | 81.61 ± 16.66 | 82.17 ± 16.41 | 81.37 ± 16.77 | t=1.17 | 0.240 |
| Temperature, ℃, Mean ± SD | 36.83 ± 0.68 | 36.85 ± 0.70 | 36.81 ± 0.67 | t=1.38 | 0.168 |
| SPO_2_, %, Mean ± SD | 97.74 ± 3.44 | 97.71 ± 3.34 | 97.75 ± 3.48 | t=-0.24 | 0.809 |
| SAPS Ⅱ score, M (Q_1_, Q_3_) | 33.00 (26.00, 41.00) | 33.00 (26.00, 41.00) | 33.00 (26.00, 41.00) | Z=1.088 | 0.277 |
| SOFA score, M (Q_1_, Q_3_) | 3.00 (2.00, 5.00) | 4.00 (2.00, 5.00) | 3.00 (2.00, 5.00) | Z=0.162 | 0.871 |
| qSOFA score, M (Q_1_, Q_3_) | 2.00 (1.00, 2.00) | 2.00 (1.00, 3.00) | 2.00 (1.00, 2.00) | Z=1.547 | 0.122 |
| GCS, M (Q_1_, Q_3_) | 12.45 ± 3.33 | 12.38 ± 3.43 | 12.48 ± 3.29 | t=-0.72 | 0.470 |
| CCI, M (Q_1_, Q_3_) | 4.00 (2.00, 6.00) | 4.00 (2.00, 6.00) | 4.00 (3.00, 6.00) | Z=0.127 | 0.899 |
| WBC, K/uL, M (Q_1_, Q_3_) | 10.40 (8.00, 13.30) | 10.50 (8.00, 13.20) | 10.40 (8.00, 13.30) | Z=0.188 | 0.851 |
| Hemoglobin, g/dL, Mean ± SD | 12.05 ± 2.00 | 12.06 ± 2.00 | 12.05 ± 2.00 | t=0.15 | 0.877 |
| Hematocrit, %, Mean ± SD | 35.83 ± 5.73 | 35.91 ± 5.78 | 35.79 ± 5.70 | t=0.50 | 0.619 |
| Creatinine, mg/dL, M (Q_1_, Q_3_) | 0.90 (0.70, 1.10) | 0.90 (0.70, 1.10) | 0.90 (0.70, 1.10) | Z=0.414 | 0.679 |
| INR, ratio, M (Q_1_, Q_3_) | 1.13 (1.10, 1.30) | 1.13 (1.10, 1.30) | 1.13 (1.10, 1.20) | Z=0.019 | 0.985 |
| Prothrombin time, sec, Mean ± SD | 12.78 (12.00, 13.90) | 12.80 (12.00, 14.10) | 12.76 (12.00, 13.90) | Z=0.816 | 0.414 |
| Blood urea nitrogen, mg/dL, M (Q_1_, Q_3_) | 16.00 (12.00, 22.00) | 16.00 (12.00, 22.00) | 16.00 (12.00, 22.00) | Z=-0.235 | 0.814 |
| Glucose, mg/dL, M (Q_1_, Q_3_) | 133.00 (111.00, 165.00) | 134.00 (112.00, 168.00) | 133.00 (111.00, 163.00) | Z=1.755 | 0.079 |
| Bicarbonate, mEq/L, Mean ± SD | 23.85 ± 3.64 | 23.87 ± 3.50 | 23.84 ± 3.70 | t=0.20 | 0.841 |
| Sodium, mEq/L, Mean ± SD | 139.11 ± 4.71 | 139.13 ± 4.82 | 139.10 ± 4.67 | t=0.14 | 0.887 |
| Potassium, mEq/L, Mean ± SD | 3.94 ± 0.66 | 3.91 ± 0.60 | 3.95 ± 0.68 | t=-1.68 | 0.094 |
| Chloride, mEq/L, Mean ± SD | 103.70 ± 5.37 | 103.71 ± 5.35 | 103.70 ± 5.38 | t=0.06 | 0.951 |
| Urine output, mL, M (Q_1_, Q_3_) | 1740.00 (1153.00, 2475.00) | 1755.00 (1138.00, 2565.00) | 1730.00 (1170.00, 2441.00) | Z=0.669 | 0.504 |
| Mannitol, n (%) |  |  |  | χ^2^=1.767 | 0.184 |
| No | 2266 (80.27) | 667 (78.75) | 1599 (80.92) |  |  |
| Yes | 557 (19.73) | 180 (21.25) | 377 (19.08) |  |  |
| Anticoagulation, n (%) |  |  |  | χ^2^=0.805 | 0.370 |
| No | 2177 (77.12) | 644 (76.03) | 1533 (77.58) |  |  |
| Yes | 646 (22.88) | 203 (23.97) | 443 (22.42) |  |  |
| Blood transfusion, n (%) |  |  |  | χ^2^=0.010 | 0.922 |
| No | 2223 (78.75) | 666 (78.63) | 1557 (78.80) |  |  |
| Yes | 600 (21.25) | 181 (21.37) | 419 (21.20) |  |  |
| Surgery, n (%) |  |  |  | χ^2^=0.031 | 0.985 |
| Craniotomy | 81 (2.87) | 25 (2.95) | 56 (2.83) |  |  |
| Minimally invasive surgery | 251 (8.89) | 75 (8.85) | 176 (8.91) |  |  |
| No surgery | 2491 (88.24) | 747 (88.19) | 1744 (88.26) |  |  |
| Neurodegeneration, n (%) |  |  |  | χ^2^=0.273 | 0.601 |
| No | 1522 (53.91) | 463 (54.66) | 1059 (53.59) |  |  |
| Yes | 1301 (46.09) | 384 (45.34) | 917 (46.41) |  |  |
| Length of hospital stay, days, M (Q_1_, Q_3_) | 3.76 (2.01, 7.94) | 3.83 (2.04, 7.80) | 3.73 (1.99, 8.03) | Z=0.877 | 0.380 |
| ICU stay, days, M (Q_1_, Q_3_) | 3.99 (2.08, 8.65) | 4.07 (2.13, 8.17) | 3.92 (2.05, 8.90) | Z=0.655 | 0.513 |
| ICU status, n (%) |  |  |  | χ^2^=0.100 | 0.951 |
| ICU death | 96 (3.40) | 30 (3.54) | 66 (3.34) |  |  |
| ICU discharge | 2446 (86.65) | 734 (86.66) | 1712 (86.64) |  |  |
| ICU readmission | 281 (9.95) | 83 (9.80) | 198 (10.02) |  |  |
| Survival time within 30-day, days, M (Q_1_, Q_3_) | 30.00 (18.16, 30.00) | 30.00 (15.32, 30.00) | 30.00 (20.10, 30.00) | Z=-1.136 | 0.256 |
| RPR, M (Q_1_, Q_3_) | 0.07 (0.05, 0.09) | 0.07 (0.05, 0.09) | 0.07 (0.05, 0.09) | Z=1.448 | 0.148 |
| RPR, n (%) |  |  |  | χ^2^=1.015 | 0.602 |
| Tertile 1 (<0.057) | 941 (33.33) | 271 (32.00) | 670 (33.91) |  |  |
| Tertile 2 (0.057-0.078) | 941 (33.33) | 286 (33.77) | 655 (33.15) |  |  |
| Tertile 3 (>0.078) | 941 (33.33) | 290 (34.24) | 651 (32.95) |  |  |
| Survival status within 30-day, n (%) |  |  |  | χ^2^=1.252 | 0.263 |
| Survivors | 2024 (71.70) | 595 (70.25) | 1429 (72.32) |  |  |
| Non-survivors | 799 (28.30) | 252 (29.75) | 547 (27.68) |  |  |

Note: ICU, intensive care unit; MICU, medical ICU; SICU, surgical ICU; SBP, systolic blood pressure; DBP, diastolic blood pressure; SPO_2_, saturation of peripheral oxygen; SAPS Ⅱ, Simplified Acute Physiology Score; SOFA, Sequential Organ Failure Assessment; qSOFA, quick SOFA; GCS, Glasgow Coma Score; CCI, Charlson comorbidity index; WBC, white blood cell; INR, international normalized ratio; RPR, red cell distribution width to platelet ratio.

**Supplement Table 2**. Competitive risk analysis of factors associated with patient readmission to the ICU.

| **Variables** | **Univariate analysis** | | **Multivariate analysis** | |
| --- | --- | --- | --- | --- |
|  | HR (95%CI) | *P* | HR (95%CI) | *P* |
| Age | 1.00 (0.99-1.01) | 0.726 |  |  |
| Sex |  |  |  |  |
| Female | Ref |  | Ref |  |
| Male | 1.39 (1.09-1.77) | 0.008 | 1.36 (1.05-1.75) | 0.020 |
| Race |  |  |  |  |
| Black | Ref |  | Ref |  |
| Others | 2.03 (1.14-3.60) | 0.016 | 2.09 (1.18-3.68) | 0.011 |
| Unknown | 1.55 (0.88-2.71) | 0.126 | 1.61 (0.90-2.85) | 0.106 |
| White | 2.38 (1.45-3.90) | <.001 | 2.16 (1.31-3.57) | 0.002 |
| Insurance |  |  |  |  |
| Medicare | Ref |  |  |  |
| Others | 1.11 (0.87-1.43) | 0.398 |  |  |
| Private | 0.92 (0.62-1.37) | 0.682 |  |  |
| Admission type |  |  |  |  |
| Emergency | Ref |  |  |  |
| Non-emergency | 1.23 (0.95-1.58) | 0.117 |  |  |
| ICU type |  |  |  |  |
| MICU | Ref |  |  |  |
| Others | 0.73 (0.45-1.19) | 0.206 |  |  |
| SICU | 0.74 (0.54-1.00) | 0.051 |  |  |
| Ventilation |  |  |  |  |
| No | Ref |  | Ref |  |
| Yes | 0.29 (0.22-0.38) | <0.001 | 0.53 (0.39-0.73) | <0.001 |
| Vasopressor |  |  |  |  |
| No | Ref |  | Ref |  |
| Yes | 0.36 (0.28-0.48) | <0.001 | 0.51 (0.38-0.69) | <0.001 |
| Renal replacement therapy |  |  |  |  |
| No | Ref |  |  |  |
| Yes | 1.03 (0.65-1.62) | 0.915 |  |  |
| Congestive heart failure |  |  |  |  |
| No | Ref |  |  |  |
| Yes | 1.02 (0.75-1.40) | 0.899 |  |  |
| Sepsis |  |  |  |  |
| No | Ref |  | Ref |  |
| Yes | 0.26 (0.20-0.35) | <0.001 | 0.36 (0.27-0.48) | <0.001 |
| Atrial fibrillation |  |  |  |  |
| No | Ref |  |  |  |
| Yes | 1.07 (0.80-1.44) | 0.641 |  |  |
| Diabetes |  |  |  |  |
| No | Ref |  |  |  |
| Yes | 0.85 (0.64-1.12) | 0.235 |  |  |
| SBP | 0.99 (0.99-0.99) | 0.041 | 0.99 (0.99-0.99) | 0.029 |
| DBP | 1.00 (0.99-1.01) | 0.841 |  |  |
| Respiratory rate | 1.00 (0.97-1.03) | 0.954 |  |  |
| Heart rate | 1.00 (1.00-1.01) | 0.338 |  |  |
| Temperature | 0.86 (0.72-1.02) | 0.091 |  |  |
| SPO_2_ | 0.98 (0.95-1.01) | 0.128 |  |  |
| CCI | 1.06 (1.01-1.10) | 0.013 | 0.99 (0.93-1.05) | 0.740 |
| WBC | 0.98 (0.94-1.02) | 0.287 |  |  |
| Hemoglobin | 0.95 (0.90-1.00) | 0.057 |  |  |
| Hematocrit | 0.98 (0.96-0.99) | 0.027 | 0.97 (0.95-0.99) | 0.004 |
| Creatinine | 1.02 (0.98-1.06) | 0.343 |  |  |
| INR | 1.06 (0.91-1.24) | 0.444 |  |  |
| Prothrombin time | 1.02 (1.01-1.03) | 0.026 | 1.02 (1.01-1.04) | 0.017 |
| Blood urea nitrogen | 1.00 (1.00-1.01) | 0.503 |  |  |
| Glucose | 0.99 (0.99-0.99) | 0.006 | 1.00 (1.00-1.00) | 0.204 |
| Bicarbonate | 1.03 (1.01-1.06) | 0.046 | 1.01 (0.98-1.05) | 0.447 |
| Sodium | 0.98 (0.96-1.01) | 0.149 |  |  |
| Potassium | 1.05 (0.87-1.26) | 0.596 |  |  |
| Chloride | 1.00 (0.98-1.02) | 0.979 |  |  |
| Urine output | 1.00 (1.00-1.00) | 0.075 |  |  |
| Mannitol |  |  |  |  |
| No | Ref |  | Ref |  |
| Yes | 0.53 (0.39-0.71) | <0.001 | 0.70 (0.50-0.98) | 0.038 |
| Anticoagulation |  |  |  |  |
| No | Ref |  |  |  |
| Yes | 0.85 (0.67-1.08) | 0.177 |  |  |
| Blood transfusion |  |  |  |  |
| No | Ref |  | Ref |  |
| Yes | 0.54 (0.41-0.71) | <0.001 | 0.64 (0.47-0.89) | 0.007 |
| Surgery |  |  |  |  |
| Craniotomy | Ref |  | Ref |  |
| Minimally invasive surgery | 1.89 (0.94-3.78) | 0.074 | 0.91 (0.43-1.96) | 0.814 |
| No surgery | 2.51 (1.32-4.78) | 0.005 | 1.28 (0.64-2.59) | 0.488 |
| Neurodegeneration |  |  |  |  |
| No | Ref |  | Ref |  |
| Yes | 0.73 (0.58-0.92) | 0.008 | 0.70 (0.55-0.91) | 0.006 |
| RPR |  |  |  |  |
| Tertile 1 (<0.057) | 1.36 (1.01-1.85) | 0.048 | 1.45 (1.07-1.97) | 0.017 |
| Tertile 2 (0.057-0.078) | Ref |  | Ref |  |
| Tertile 3 (>0.078) | 1.63 (1.21-2.19) | 0.001 | 1.56 (1.13-2.15) | 0.006 |

Note: ICU, intensive care unit; MICU, medical ICU; SICU, surgical ICU; SBP, systolic blood pressure; DBP, diastolic blood pressure; SPO2, saturation of peripheral oxygen; CCI, Charlson comorbidity index; WBC, white blood cell; INR, international normalized ratio; RPR, red cell distribution width to platelet ratio.
