# Supplementary material for: Predictive value of admission red cell distribution width-to-platelet ratio for 30-day death in patients with spontaneous intracerebral hemorrhage: an analysis of the MIMIC database
Source: Front Neurol. 2023 Oct 18;14:1221335. doi: 10.3389/fneur.2023.1221335 (PMC10618669; doi:10.3389/fneur.2023.1221335)
Supplement: Supplementary file 5 [file Data_Sheet_4.DOCX]

# Data preprocessing：

import pandas as pd # 数据表结构处理

import numpy as np # 数据结构处理

# from my_scipy import stats as ms#输出AUC值等

from my_scipy import dataprocessing as mp#查看缺失值

import re

from scipy import stats

import metrics1_v2

# from my_scipy import metrics

import matplotlib.pyplot as plt

from datetime import datetime

from sklearn.metrics import accuracy_score,roc_curve

from metrics1_v2 import model_parameter

from sklearn.preprocessing import StandardScaler

from sklearn.calibration import calibration_curve

pd.set_option('display.max_rows',150)

# 加载数据

data_ori = pd.read_csv("Row_dataV3.csv")

data_ori

# 变量名

print(data_ori.columns.tolist())

# 查看缺失值

mp.var_select(data_ori)

# 查看重复值

data_ori.duplicated().sum()

data0 = data_ori.copy()

data0.shape

# 纳入规则1：

# aged≥18 years old

data1 = data0[(data0["age"] >= 18) & (data0["age"] < 90)].copy()

data1.shape

# 纳入规则2：

# hospitalized in the ICU for at least 24 hours

data2 = data1[data1["los"] >= 1.0].copy()

data2.shape

# 排除规则1

# missing key data, such as RDW, platelet

data3 = data2.dropna(subset = (["rdw", "platelet"]), axis = 0)

data3.shape

# 查看RDW, platelet的分布情况

mp.var_review(data3[['rdw', 'platelet']])

# 排除规则2：

# missing survival information.

data4 = data3.copy()

# 30天死亡时间

data4["intime"] = data4["intime"].apply(pd.to_datetime,format='%Y-%m-%d %H:%M:%S')

data4["dod"] = data4["dod"].apply(pd.to_datetime,format='%Y-%m-%d %H:%M:%S')

data4["dischtime"] = data4["dischtime"].apply(pd.to_datetime,format='%Y-%m-%d %H:%M:%S')

data4["Time30"] = (data4["dod"] - data4["intime"]).map(lambda x:x.days) + (data4["dod"] - data4["intime"]).map(lambda x:x.seconds)/3600/24.0

for i in data4.index:

if data4.loc[i, "expire_flag"] == 1:

if data4.loc[i, "Time30"] < 30.0:

data4.loc[i, "Time30"] = data4.loc[i, "Time30"]

data4.loc[i, "expire_flag30"] = 1

else:

data4.loc[i, "Time30"] = 30.0

data4.loc[i, "expire_flag30"] = 0

else:

if data4.loc[i, "Time30"] < 30.0:

data4 = data4.drop(columns = ["Time30"], axis = 0)

else:

data4.loc[i, "Time30"] = 30.0

data4.loc[i, "expire_flag30"] = 0

mp.var_review(data4[["Time30", "expire_flag30"]])

# data4 = data4[data4["Time"] > 0]

# data4.shape

# 15天死亡时间

data4["intime"] = data4["intime"].apply(pd.to_datetime,format='%Y-%m-%d %H:%M:%S')

data4["dod"] = data4["dod"].apply(pd.to_datetime,format='%Y-%m-%d %H:%M:%S')

data4["dischtime"] = data4["dischtime"].apply(pd.to_datetime,format='%Y-%m-%d %H:%M:%S')

data4["Time15"] = (data4["dod"] - data4["intime"]).map(lambda x:x.days) + (data4["dod"] - data4["intime"]).map(lambda x:x.seconds)/3600/24.0

for i in data4.index:

if data4.loc[i, "expire_flag"] == 1:

if data4.loc[i, "Time15"] < 15.0:

data4.loc[i, "Time15"] = data4.loc[i, "Time15"]

data4.loc[i, "expire_flag15"] = 1

else:

data4.loc[i, "Time15"] = 15.0

data4.loc[i, "expire_flag15"] = 0

else:

if data4.loc[i, "Time15"] < 15.0:

data4 = data4.drop(columns = ["Time15"], axis = 0)

else:

data4.loc[i, "Time15"] = 15.0

data4.loc[i, "expire_flag15"] = 0

mp.var_review(data4[["Time15", "expire_flag15"]])

# data4 = data4[data4["Time"] > 0]

# data4.shape

data4.shape

print("开始一共有{}行{}列样本".format(data0.shape[0], data0.shape[1]))

print("=============================")

for i in range(1,5):

if i>0:

exec("temp1 = data{}".format(i-1))

exec("temp2 = data{}".format(i))

t1 = temp2.shape[0]

t2 = temp2.shape[1]

a = temp1.shape[0]-temp2.shape[0]

if i<3:

print("根据纳入标准第{}条，排除{}人".format(i,a))

print(" 还剩下一共{}行{}列样本".format(t1, t2))

else:

print("根据排除标准第{}条，排除{}人".format(i-2,a))

print(" 还剩下一共{}行{}列样本".format(t1, t2))

print("=============================")

print("还剩下一共有{}行{}列样本".format(data4.shape[0], data4.shape[1]))

# 缺失值统计

# 查看缺失值

datanew1 = data4.copy()

mp.var_select(datanew1)

mp.var_select(datanew1).to_excel("总缺失数统计.xlsx")

print(mp.var_select(datanew1)["缺失值百分比"].index.tolist())

# 筛选出缺失值比例≤20%的变量

data_1 = mp.var_select(datanew1)

list_a = [kk for kk in datanew1.columns if kk in data_1[data_1.缺失值百分比.str.replace("%","").apply(float)<=20].index.tolist()]

for kk in ['expire_flag', 'hospital_expire_flag', 'outtime', 'intime', 'last_careunit',

'dischtime', 'subject_id', 'admittime', 'hadm_id', 'flag', 'respiratory_rate_unit'

]:

list_a.remove(kk)

list_a

# 重新赋值

datanew2 = datanew1.loc[:,list_a].copy()

datanew2

# 缺失值统计

# 查看缺失值

mp.var_select(datanew2)

# 主要研究因素 % / K/uL=KuL

datanew2["RPR"] = (datanew2["rdw"] / datanew2["platelet"])

mp.var_review(datanew2[["RPR"]])

# 生存信息

mp.var_review(datanew2[["Time30", "expire_flag30"]])

# 再入ICU

mp.var_review(datanew2[['icu_status', 'icu_follow_time']])

# 变量名

datanew3 = datanew2.copy()

print(datanew3.columns.tolist())

# age

# mp.abnormal_value_find(datanew3[["age"]])

mp.var_review(datanew3[["age"]])

# gender

datanew3.loc[:,"gender"] = datanew3.loc[:,"gender"].apply(lambda x: 'Male' if x == 'M' else ('Female' if x == 'F' else np.nan))

mp.var_review(datanew3[["gender"]])

# ethnicity

datanew3.loc[:,"ethnicity"] = datanew3.loc[:,"ethnicity"].apply(lambda x: "White" if "WHITE" in str(x) else ("Black" if "BLACK" in str(x) else ("Unknown" if "UNKNOWN" in str(x) else "Others")))

mp.var_review(datanew3[["ethnicity"]])

# insurance

datanew3.loc[:,"insurance"] = datanew3.loc[:,"insurance"].apply(lambda x: "Private" if "Private" in str(x) else ("Medicare" if "Medicare" in str(x) else "Others"))

mp.var_review(datanew3[["insurance"]])

# admission_type

datanew3.loc[:,"admission_type"] = datanew3.loc[:,"admission_type"].apply(lambda x: "EMERGENCY" if "EMERGENCY" in str(x) else "NO_EMERGENCY")

mp.var_review(datanew3[["admission_type"]])

# first_careunit

datanew3.loc[:,"first_careunit"] = datanew3.loc[:,"first_careunit"].apply(lambda x: "MICU" if "MICU" in str(x) else ("SICU" if "SICU" in str(x) else "Others"))

mp.var_review(datanew3[["first_careunit"]])

# Ventilation use、Vasopressor use、Renal replacement therapy

datanew3[['isvent', 'isvaso', 'isrrt']] = datanew3[['isvent', 'isvaso', 'isrrt']].applymap(lambda x: "Yes" if x == 1 else("No" if x ==0 else np.nan))

mp.var_review(datanew3[['isvent', 'isvaso', 'isrrt']])

# surgery (craniotomy/minimally invasive surgery/no-surgery)'craniotomy', 'minimally_invasive_surgery'

for i in datanew3.index:

if datanew3.loc[i, "craniotomy"] == 1:

datanew3.loc[i, "surgery"] = "craniotomy"

elif datanew3.loc[i, "minimally_invasive_surgery"] == 1:

datanew3.loc[i, "surgery"] = "minimally_invasive_surgery"

else:

datanew3.loc[i, "surgery"] = "no_surgery"

datanew3['mannitol'] = datanew3['mannitol'].apply(lambda x: "Yes" if x == 1 else("No" if x ==0 else np.nan))

mp.var_review(datanew3[['surgery', 'mannitol']])

# comorbidities (congestive heart failure, atrial fibrillation, diabetes, hypertension, malignancy)

datanew3["diabetes"] = datanew3["diabetes_without_cc"] + datanew3["diabetes_with_cc"]

datanew3.loc[:,"diabetes"] = datanew3.loc[:,"diabetes"].apply(lambda x: 1 if x > 0 else (0 if x == 0 else np.nan))

datanew3[['diabetes', 'congestive_heart_failure', 'atrial_fibrillation', 'hypertension', 'malignant_cancer', 'sepsis']] = datanew3[['diabetes', 'congestive_heart_failure', 'atrial_fibrillation', 'hypertension', 'malignant_cancer', 'sepsis']].applymap(lambda x: "Yes" if x == 1 else("No" if x ==0 else np.nan))

mp.var_review(datanew3[['diabetes', 'congestive_heart_failure', 'atrial_fibrillation', 'hypertension', 'malignant_cancer', 'sepsis']])

# 生命特征指标

mp.abnormal_value_find(datanew3[['heart_rate', 'systolic', 'diastolic', 'temperature', 'respiratory_rate']])

mp.var_review(datanew3[['heart_rate', 'systolic', 'diastolic', 'temperature', 'respiratory_rate']])

datanew3[['heart_rate', 'systolic', 'diastolic', 'temperature', 'respiratory_rate']].describe()

lower_heart_rate = np.nanmean(datanew3["heart_rate"]) - 3 * np.nanstd(datanew3["heart_rate"])

upper_heart_rate = np.nanmean(datanew3["heart_rate"]) + 3 * np.nanstd(datanew3["heart_rate"])

lower_systolic = np.nanmean(datanew3["systolic"]) - 3 * np.nanstd(datanew3["systolic"])

upper_systolic = np.nanmean(datanew3["systolic"]) + 3 * np.nanstd(datanew3["systolic"])

lower_diastolic = np.nanmean(datanew3["diastolic"]) - 3 * np.nanstd(datanew3["diastolic"])

upper_diastolic = np.nanmean(datanew3["diastolic"]) + 3 * np.nanstd(datanew3["diastolic"])

lower_temperature = np.nanmean(datanew3["temperature"]) - 3 * np.nanstd(datanew3["temperature"])

upper_temperature = np.nanmean(datanew3["temperature"]) + 3 * np.nanstd(datanew3["temperature"])

lower_respiratory_rate = np.nanmean(datanew3["respiratory_rate"]) - 3 * np.nanstd(datanew3["respiratory_rate"])

upper_respiratory_rate = np.nanmean(datanew3["respiratory_rate"]) + 3 * np.nanstd(datanew3["respiratory_rate"])

d = {'value': {

'lower_heart_rate':lower_heart_rate, 'upper_heart_rate':upper_heart_rate,

'lower_systolic':lower_systolic, 'upper_systolic':upper_systolic,

'lower_diastolic':lower_diastolic, 'upper_diastolic':upper_diastolic,

'lower_temperature':lower_temperature, 'upper_temperature':upper_temperature,

'lower_respiratory_rate':lower_respiratory_rate, 'upper_respiratory_rate':upper_respiratory_rate}}

pd.DataFrame.from_dict(d, orient = 'columns')

# 异常值处理

datanew3.loc[:,'heart_rate'] = datanew3.loc[:,'heart_rate'].apply(lambda x: np.nan if ((x <= np.nanmean(datanew3["heart_rate"]) - 3 * np.nanstd(datanew3["heart_rate"])) | (x >= np.nanmean(datanew3["heart_rate"]) + 3 * np.nanstd(datanew3["heart_rate"]))) else x)

datanew3.loc[:,'systolic'] = datanew3.loc[:,'systolic'].apply(lambda x: np.nan if ((x <= np.nanmean(datanew3["systolic"]) - 3 * np.nanstd(datanew3["systolic"])) | (x >= np.nanmean(datanew3["systolic"]) + 3 * np.nanstd(datanew3["systolic"]))) else x)

datanew3.loc[:,'diastolic'] = datanew3.loc[:,'diastolic'].apply(lambda x: np.nan if ((x <= 0) | (x >= np.nanmean(datanew3["diastolic"]) + 3 * np.nanstd(datanew3["diastolic"]))) else x)

datanew3.loc[:,'temperature'] = datanew3.loc[:,'temperature'].apply(lambda x: np.nan if ((x <= np.nanmean(datanew3["temperature"]) - 3 * np.nanstd(datanew3["temperature"])) | (x >= np.nanmean(datanew3["temperature"]) + 3 * np.nanstd(datanew3["temperature"]))) else x)

datanew3.loc[:,'respiratory_rate'] = datanew3.loc[:,'respiratory_rate'].apply(lambda x: np.nan if ((x <= np.nanmean(datanew3["respiratory_rate"]) - 3 * np.nanstd(datanew3["respiratory_rate"])) | (x >= np.nanmean(datanew3["respiratory_rate"]) + 3 * np.nanstd(datanew3["respiratory_rate"]))) else x)

mp.var_review(datanew3[['systolic', 'diastolic', 'respiratory_rate', 'heart_rate', 'temperature']])

datanew3[['systolic', 'diastolic', 'respiratory_rate', 'heart_rate', 'temperature']].describe()

# Laboratory parameters(Blood)

mp.abnormal_value_find(datanew3[['wbc', 'platelet', 'hemoglobin', 'rdw', 'hematocrit', 'creatinine_blood', 'inr', 'pt_lab', 'bun', 'glucose_lab', 'bicarbonate', 'sodium', 'potassium', 'chloride', 'spo2']])

datanew3[['wbc', 'platelet', 'hemoglobin', 'rdw', 'hematocrit', 'creatinine_blood', 'inr', 'pt_lab', 'bun', 'glucose_lab', 'bicarbonate', 'sodium', 'potassium', 'chloride', 'spo2']].describe()

mp.var_review(datanew3[['wbc', 'platelet', 'hemoglobin', 'rdw', 'hematocrit', 'creatinine_blood', 'inr', 'pt_lab', 'bun', 'glucose_lab', 'bicarbonate', 'sodium', 'potassium', 'chloride', 'spo2']])

# Scores

mp.var_review(datanew3[['sapsii', 'sofa', 'qsofa', 'gcs', 'charlson_comorbidity_index']])

# others

datanew3['anticoagulation'] = ['Yes' if x == 1 else('No' if x == 0 else None) for x in datanew3['anticoagulation']]

datanew3['neurodegeneration'] = ['Yes' if x == 1 else('No' if x == 0 else None) for x in datanew3['gcs_48h']]

datanew3['blood_transfusion'] = ['Yes' if x == 1 else('No' if x == 0 else None) for x in datanew3['blood_transfusion']]

mp.var_review(datanew3[['los', 'urine_output', 'mannitol', 'antiplatelet', 'anticoagulation', 'neurodegeneration', 'blood_transfusion']])

datanew4 = datanew3.copy()

datanew4 = datanew4.drop(columns = ["diabetes_without_cc", "diabetes_with_cc", 'craniotomy', 'minimally_invasive_surgery',

'platelet', "rdw", 'antiplatelet', 'gcs_48h'])

# 查看变量名

print(datanew4.columns.tolist())

datanew4.columns = ['Icustay_id', 'Los', 'First_careunit', 'Age', 'Gender', 'Ethnicity', 'Insurance', 'Admission_type', 'Congestive_heart_failure', 'Sepsis', 'Atrial_fibrillation', 'Hypertension', 'Malignant_cancer', 'Ventilation', 'Vasopressor', 'Rrt', 'Heart_rate', 'Systolic', 'Diastolic', 'Respiratory_rate', 'Temperature', 'Sofa', 'qsofa', 'Sapsii', 'GCS', 'Charlson_comorbidity_index', 'WBC', 'Hemoglobin', 'Hematocrit', 'Creatinine', 'INR', 'PT', 'BUN', 'Glucose', 'Bicarbonate', 'Sodium', 'Potassium', 'Chloride', 'SPO2', 'Urine_output', 'Mannitol', 'ICU_status', 'ICU_follow_time', 'Anticoagulation', 'blood_transfusion', 'Time30', 'Expire_flag30', 'Time15', 'Expire_flag15', 'RPR', 'Surgery', 'Diabetes', 'neurodegeneration']

mp.var_select(datanew4)

mp.var_select(datanew4).to_excel("缺失值统计表.xlsx")

datanew5 = datanew4.copy()

datanew5.to_csv("Impute_before.csv", index = False) #导出插补前数据

# 连续变量和分类变量筛选

list_col = mp.class_find(datanew5, list_co =['qsofa'])

list_col

datanew5.loc[:,list_col[1]]

# 数据标准化

ss = StandardScaler()

datanew5.loc[:,list_col[1]] = ss.fit_transform(datanew5.loc[:,list_col[1]]).copy()

# 数据插补

data_new_nomissing = mp.random_forest_imputation(datanew5,list_class = list_col[0],list_continuous=list_col[1],effectiveness_evaluation = True,ee_y='Expire_flag30',ee_scoring='accuracy',n_jobs_=-1,random_state_=20221001)

data_new_nomissing

# 数据标准化还原

data_new_nomissing.loc[:,list_col[1]] = ss.inverse_transform(data_new_nomissing.loc[:,list_col[1]])

data_new_nomissing

# 插补前数据还原

datanew5.loc[:,list_col[1]] = ss.inverse_transform(datanew5.loc[:,list_col[1]])

datanew5

mp.class_find(data_new_nomissing, list_co =['qsofa'])

data_new_nomissing.to_csv("Impute_after.csv", index = False)

missing_info = mp.var_select(datanew5)

list_col = [kk for kk in datanew5.columns if kk in missing_info[(missing_info.缺失值百分比.str.replace("%","").apply(float)<=20) & (missing_info.缺失值百分比.str.replace("%","").apply(float)>0)].index.tolist()]

print(f'{len(list_col)}个缺失变量，分别为：{list_col}')

# 敏感性分析

data_1 = datanew5[list_col].copy();data_2 = data_new_nomissing[list_col].copy();data_1.loc[:,'group'] = '插补前';data_2.loc[:,'group'] = '插补后'

data_mg = pd.concat([data_1,data_2],axis = 0).copy()

data_mg.to_excel("Impute.xlsx")

# data_mg_base = ms.baseline_analysis_varrecover(data_mg,'group',list_class = mp.class_find(data_mg, list_co =['qsofa'])[0],list_group = ['插补前','插补后'],fisher_=True)

# data_mg_base

data_final = data_new_nomissing.copy().reset_index(drop=True)

plt.hist(data_final["RPR"], bins = 20)

Q1 = np.percentile(data_final["RPR"], 100/3)

Q2 = np.percentile(data_final["RPR"], 200/3)

Q3 = np.percentile(data_final["RPR"], 300/3)

Q4 = np.percentile(data_final["RPR"], 400/4)

Q5 = np.percentile(data_final["RPR"], 500/6)

print("Q1为{}，Q2为{}，Q3为{}，Q4为{}, Q5为{}".format(Q1,Q2,Q3,Q4,Q5))

data_final.loc[:, "RPR_CAT"] = data_final.loc[:, "RPR"].apply(lambda x: '1' if x <= Q1 else

('2' if (x > Q1 and x <= Q2) else '3' ))

# data_final.loc[:, "RPR_CAT"] = data_final.loc[:, "RPR"].apply(lambda x: '1' if x <= Q2 else '2' )

mp.var_review(data_final[['RPR_CAT']])

data_final.to_excel("analysis_data.xlsx")

print(data_final.columns.tolist())

from sklearn.model_selection import train_test_split

unique_name = data_final["Icustay_id"].unique().tolist()

features_X_train, features_X_test, target_train, target_test = train_test_split(unique_name, unique_name, test_size=0.3, random_state=42, shuffle=True)

print(len(features_X_train), len(features_X_test), len(unique_name))

features_X_train_index = data_final[data_final['Icustay_id'].isin(features_X_train)].index.tolist()

features_X_test_index = data_final[data_final['Icustay_id'].isin(features_X_test)].index.tolist()

train_dataset = data_final.iloc[features_X_train_index]

test_dataset = data_final.iloc[features_X_test_index]

print(f'全集维度为：{data_final.shape}，训练集维度：{train_dataset.shape}，测试集维度：{test_dataset.shape}')

train_dataset.to_csv("train_dataset.csv", encoding="utf_8_sig", index=False)

test_dataset.to_csv("test_dataset.csv", encoding="utf_8_sig", index=False)

# Descriptive statistical analysis：

*加载包;

%let dir=%sysfunc(prxchange(s/(.*)\\.*/\1/,-1,%upcase(%sysget(sas_execfilepath))));

%inc "F:\SAS\ph_reg_v7.sas";

%inc "Z:\sas_code\useful\import.sas";

%inc "F:\SAS\data_statistic_sv5.sas";

*导入插补前后数据集;

**proc** **import** dbms = xlsx out= impute replace

datafile = "&dir\Impute.xlsx";

**run**;

*插补前后敏感性分析;

%***data_stastic***(data=impute,

var_list = Heart_rate|**2**\Systolic|**2**\Diastolic|**2**\Respiratory_rate|**2**\Temperature|**2**\Creatinine|**2**\INR|**2**\PT|**2**\BUN|**2**\Glucose|**2**\Bicarbonate|**2**\Sodium|**2**\Potassium|**2**\

Chloride|**2**\SPO2|**2**\Urine_output|**2**\gcs|**2**\,

group=group,

all_outcome=table1,dec=**3**,

rtf_out=**1**,rtf_a4=**0**,

dir=&dir\插补前后敏感性分析.rtf);

*导入数据（一般用于规整数据导入csv文件）;

/*全数据集*/

**proc** **import** dbms=xlsx out=all_data replace

datafile="&dir\analysis_data.xlsx";

**run**;

/*训练集*/

**proc** **import** dbms=csv out=train_data replace

datafile="&dir\train_dataset.csv"; guessingrows=max;

**run**;

/*测试集*/

**proc** **import** dbms=csv out=test_data replace

datafile="&dir\test_dataset.csv"; guessingrows=max;

**run**;

**data** all_data;

set train_data(in=a) test_data(in=b);

if a then label="Train_dataset";

else if b then label="Test_dataset";

**run**;

*组间差异性分析-全集;

%***data_stastic***(data=all_data,

var_list=Age|**2**\Gender|**1**\Ethnicity|**1**\Insurance|**1**\Admission_type|**1**\First_careunit|**1**\Ventilation|**1**\Vasopressor|**1**\Rrt|**1**\Congestive_heart_failure|**1**\Sepsis|**1**\

Atrial_fibrillation|**1**\Hypertension|**1**\Malignant_cancer|**1**\Diabetes|**1**\Systolic|**2**\Diastolic|**2**\Respiratory_rate|**2**\Heart_rate|**2**\Temperature|**2**\SPO2|**2**\Sapsii|**2**\Sofa|**2**\qsofa|**2**\GCS|**2**\

Charlson_comorbidity_index|**2**\WBC|**2**\Hemoglobin|**2**\Hematocrit|**2**\Creatinine|**2**\INR|**2**\PT|**2**\BUN|**2**\Glucose|**2**\Bicarbonate|**2**\Sodium|**2**\Potassium|**2**\Chloride|**2**\Urine_output|**2**\

Mannitol|**1**\Anticoagulation|**1**\blood_transfusion|**1**\Surgery|**1**\neurodegeneration|**1**\Los|**2**\ICU_follow_time|**2**\ICU_status|**1**\Time30|**2**\RPR|**2**\RPR_CAT|**1**\,

group=Expire_flag30,

all_outcome=table2,dec=**3**,

rtf_out=**1**,rtf_a4=**0**,

dir=&dir\组间差异性分析-全集.rtf);

*组间差异性分析-训练集;

%***data_stastic***(data=train_data,

var_list=Age|**2**\Gender|**1**\Ethnicity|**1**\Insurance|**1**\Admission_type|**1**\First_careunit|**1**\Ventilation|**1**\Vasopressor|**1**\Rrt|**1**\Congestive_heart_failure|**1**\Sepsis|**1**\

Atrial_fibrillation|**1**\Hypertension|**1**\Malignant_cancer|**1**\Diabetes|**1**\Systolic|**2**\Diastolic|**2**\Respiratory_rate|**2**\Heart_rate|**2**\Temperature|**2**\SPO2|**2**\Sapsii|**2**\Sofa|**2**\qsofa|**2**\GCS|**2**\

Charlson_comorbidity_index|**2**\WBC|**2**\Hemoglobin|**2**\Hematocrit|**2**\Creatinine|**2**\INR|**2**\PT|**2**\BUN|**2**\Glucose|**2**\Bicarbonate|**2**\Sodium|**2**\Potassium|**2**\Chloride|**2**\Urine_output|**2**\

Mannitol|**1**\Anticoagulation|**1**\blood_transfusion|**1**\Surgery|**1**\neurodegeneration|**1**\Los|**2**\ICU_follow_time|**2**\ICU_status|**1**\Time30|**2**\RPR|**2**\RPR_CAT|**1**\,

group=Expire_flag30,

all_outcome=table2,dec=**3**,

rtf_out=**1**,rtf_a4=**0**,

dir=&dir\组间差异性分析-训练集.rtf);

*组间差异性分析-测试集;

%***data_stastic***(data=test_data,

var_list=Age|**2**\Gender|**1**\Ethnicity|**1**\Insurance|**1**\Admission_type|**1**\First_careunit|**1**\Ventilation|**1**\Vasopressor|**1**\Rrt|**1**\Congestive_heart_failure|**1**\Sepsis|**1**\

Atrial_fibrillation|**1**\Hypertension|**1**\Malignant_cancer|**1**\Diabetes|**1**\Systolic|**2**\Diastolic|**2**\Respiratory_rate|**2**\Heart_rate|**2**\Temperature|**2**\SPO2|**2**\Sapsii|**2**\Sofa|**2**\qsofa|**2**\GCS|**2**\

Charlson_comorbidity_index|**2**\WBC|**2**\Hemoglobin|**2**\Hematocrit|**2**\Creatinine|**2**\INR|**2**\PT|**2**\BUN|**2**\Glucose|**2**\Bicarbonate|**2**\Sodium|**2**\Potassium|**2**\Chloride|**2**\Urine_output|**2**\

Mannitol|**1**\Anticoagulation|**1**\blood_transfusion|**1**\Surgery|**1**\neurodegeneration|**1**\Los|**2**\ICU_follow_time|**2**\ICU_status|**1**\Time30|**2**\RPR|**2**\RPR_CAT|**1**\,

group=Expire_flag30,

all_outcome=table2,dec=**3**,

rtf_out=**1**,rtf_a4=**0**,

dir=&dir\组间差异性分析-测试集.rtf);

*组间差异性分析-训练集与测试集;

%***data_stastic***(data=all_data,

var_list=Age|**2**\Gender|**1**\Ethnicity|**1**\Insurance|**1**\Admission_type|**1**\First_careunit|**1**\Ventilation|**1**\Vasopressor|**1**\Rrt|**1**\Congestive_heart_failure|**1**\Sepsis|**1**\

Atrial_fibrillation|**1**\Hypertension|**1**\Malignant_cancer|**1**\Diabetes|**1**\Systolic|**2**\Diastolic|**2**\Respiratory_rate|**2**\Heart_rate|**2**\Temperature|**2**\SPO2|**2**\Sapsii|**2**\Sofa|**2**\qsofa|**2**\GCS|**2**\

Charlson_comorbidity_index|**2**\WBC|**2**\Hemoglobin|**2**\Hematocrit|**2**\Creatinine|**2**\INR|**2**\PT|**2**\BUN|**2**\Glucose|**2**\Bicarbonate|**2**\Sodium|**2**\Potassium|**2**\Chloride|**2**\Urine_output|**2**\

Mannitol|**1**\Anticoagulation|**1**\blood_transfusion|**1**\Surgery|**1**\neurodegeneration|**1**\Los|**2**\ICU_follow_time|**2**\ICU_status|**1**\Time30|**2**\RPR|**2**\RPR_CAT|**1**\Expire_flag30|**1**\,

group=label,

all_outcome=table2,dec=**3**,

rtf_out=**1**,rtf_a4=**0**,

dir=&dir\组间差异性分析-训练集与测试集.rtf);

*cox单因素分析;

*==30天死亡;

%***ph_reg***(data = all_data, m_co_var = ,

main_var = Age|**2**\Gender|**1**\Ethnicity|**1**\Insurance|**1**\Admission_type|**1**\First_careunit|**1**\Ventilation|**1**\Vasopressor|**1**\Rrt|**1**\Sepsis|**1**\

Atrial_fibrillation|**1**\Systolic|**2**\Diastolic|**2**\Respiratory_rate|**2**\Heart_rate|**2**\Temperature|**2**\SPO2|**2**\Sapsii|**2**\Sofa|**2**\qsofa|**2**\GCS|**2**\

Charlson_comorbidity_index|**2**\WBC|**2**\Hemoglobin|**2**\Hematocrit|**2**\Creatinine|**2**\INR|**2**\PT|**2**\BUN|**2**\Glucose|**2**\Bicarbonate|**2**\Sodium|**2**\Potassium|**2**\Chloride|**2**\Urine_output|**2**\

Mannitol|**1**\Anticoagulation|**1**\blood_transfusion|**1**\Surgery|**1**\neurodegeneration|**1**\RPR_CAT|**1**\,

status = Expire_flag30,time = Time30, cp = **0**, cox_model = danyinsu, output = **1**, rtf_a4 = **0**, dir = &dir.\**30**天死亡单因素.rtf);

*cox逐步回归;

*==30天死亡;

**proc** **phreg** data = all_data CONCORDANCE=HARRELL(se);

class RPR_CAT(Reference = '1') Gender Ethnicity(Reference = 'Black') Insurance Admission_type(Reference = 'EMERGENCY') First_careunit Ventilation(Reference = 'No')

Vasopressor(Reference = 'No') Rrt(Reference = 'No') Sepsis(param=ref ref='No') Atrial_fibrillation Mannitol(Reference = 'No') Surgery(Reference = 'craniotomy') blood_transfusion(param=ref ref='No')

neurodegeneration(param=ref ref='No') Anticoagulation(param=ref ref='No');

model Time30*Expire_flag30(**0**)=Age Gender Ethnicity Insurance Admission_type First_careunit Ventilation Vasopressor Rrt Sepsis Atrial_fibrillation Diastolic Respiratory_rate

Heart_rate SPO2 Charlson_comorbidity_index WBC Hemoglobin Hematocrit Creatinine INR PT BUN Glucose Bicarbonate Potassium Urine_output Mannitol Anticoagulation Surgery

blood_transfusion neurodegeneration/selection=stepwise include=**1** rl;

**quit**;*0.7631 0.0076 ;

**proc** **phreg** data = all_data CONCORDANCE=HARRELL(se);

model Time30*Expire_flag30(**0**)= Sofa/selection=stepwise include=**1** rl;

**quit**;*0.6556 0.0101 ;

**proc** **phreg** data = all_data CONCORDANCE=HARRELL(se);

model Time30*Expire_flag30(**0**)= Sapsii/selection=stepwise include=**1** rl;

**quit**;*0.7120 0.0089 ;

**proc** **phreg** data = all_data CONCORDANCE=HARRELL(se);

model Time30*Expire_flag30(**0**)= qsofa/selection=stepwise include=**1** rl;

**quit**;*0.5292 0.0094 ;

**proc** **phreg** data=all_data noprint;

class RPR_CAT(Reference = '1') Gender Ethnicity(Reference = 'Black') Insurance Admission_type(Reference = 'EMERGENCY') First_careunit Ventilation(Reference = 'No')

Vasopressor(Reference = 'No') Rrt(Reference = 'No') Sepsis(param=ref ref='No') Atrial_fibrillation Mannitol(Reference = 'No') Surgery(Reference = 'craniotomy') blood_transfusion(param=ref ref='No')

neurodegeneration(param=ref ref='No') Anticoagulation(param=ref ref='No');

model Time30*Expire_flag30(**0**)=Age Ethnicity First_careunit Ventilation Vasopressor Rrt Sepsis Heart_rate SPO2 Hemoglobin BUN Glucose Urine_output Mannitol

Anticoagulation Surgery neurodegeneration RPR_CAT ;

output out=all_data XBETA=dat1_x;

**run**;

/*单个因素的C指数比较*/

**%macro** sing_Concordance(data=,main_var=,sub=);

%let main_n=%sysfunc(countw(&main_var.,%str(" "))); /*计算主要变量的个数*/

ods html close;

%do i = **1** %to %eval(&main_n.);

%let var&i=%scan(&main_var,&i.,%str(" "));

ods output Concordance=Concordance;

proc phreg data=&data. concordance=HARRELL(SE) ;

&sub.;

class RPR_CAT(param=ref ref=first);

/* class &&var&i.(param=ref ref=first);*/

model Time30*Expire_flag30(**0**)=&&var&i. ;

quit;

data Concordance&i;

set Concordance;

length variables $32767.;

label Concordance ="%nrstr(Concordance (95%CI))";

Concordance=put(Estimate,**8.3**)||" ("||strip(put(Estimate - **1.96***StdErr,**8.3**))||" - "||strip(put(Estimate + **1.96***StdErr,**8.3**))||")";

variables="&&var&i.";

run;

%end;

data Concordance;

set Concordance1-Concordance%eval(&main_n.);

ods html;

proc print noobs label;

var variables Concordance;

run;

run;

**%mend**;

%***sing_Concordance***(data=all_data, main_var=dat1_x Sofa Sapsii qsofa,sub=);

**proc** **phreg** data=all_data concordance=uno(diff se seed=**42** iter=**500**);

model Time30*Expire_flag30(**0**)=dat1_x Sofa Sapsii qsofa/ nofit;

roc 'RPR' dat1_x;

roc 'Sofa' Sofa;

roc 'Sapsii' Sapsii;

roc 'qsofa' qsofa;

**run**;

*cox多因素分析;

*==30天死亡;

%***ph_reg***(data = all_data, m_co_var = Age|**2**\Ethnicity|**1**|Black\First_careunit|**1**\Ventilation|**1**|No\Vasopressor|**1**|No\Rrt|**1**|No\Sepsis|**1**\Heart_rate|**2**\SPO2|**2**\Hemoglobin|**2**\BUN|**2**\

Glucose|**2**\Urine_output|**2**\Mannitol|**1**|No\Anticoagulation|**1**\Surgery|**1**|craniotomy\neurodegeneration|**1**\RPR_CAT|**1**|**1**\,

main_var = ,

status = Expire_flag30,time = Time30, cp = **0**, cox_model = duoyinsu, output = **1**, rtf_a4 = **0**, dir = &dir.\**30**天死亡多因素.rtf);

* 亚组分析;

*== Age;

**data** all_data;

set all_data;

if age < **65.0** then do;

age_new = **1**;

end;

else do;

age_new = **2**;

end;

**run**;

*Model 1;

%***ph_reg***(data = all_data, m_co_var = ,

main_var = RPR_CAT|**1**|**1**\,

sub = age_new,

status = Expire_flag30,time = Time30, cp = **0**, cox_model = age1, output = **1**, rtf_a4 = **1**, dir = &dir.\Age1-**30**天死亡亚组分析.rtf);

*Model 2;

%***ph_reg***(data = all_data, m_co_var = Ethnicity|**1**|Black\Admission_type|**1**|EMERGENCY\Ventilation|**1**|No\Vasopressor|**1**|No\Rrt|**1**|No\Sepsis|**1**\Heart_rate|**2**\SPO2|**2**\Hemoglobin|**2**\BUN|**2**\

Glucose|**2**\Urine_output|**2**\Mannitol|**1**|No\Surgery|**1**|craniotomy\,

main_var = RPR_CAT|**1**|**1**\,

sub = age_new,

status = Expire_flag30,time = Time30, cp = **0**, cox_model = age2, output = **1**, rtf_a4 = **1**, dir = &dir.\Age2-**30**天死亡亚组分析.rtf);

*== Gender;

*Model 1;

%***ph_reg***(data = all_data, m_co_var = ,

main_var = RPR_CAT|**1**|**1**\,

sub = Gender,

status = Expire_flag30,time = Time30, cp = **0**, cox_model = Gender1, output = **1**, rtf_a4 = **1**, dir = &dir.\Gender1-**30**天死亡亚组分析.rtf);

*Model 2;

%***ph_reg***(data = all_data, m_co_var = Age|**2**\Ethnicity|**1**|Black\Admission_type|**1**|EMERGENCY\Ventilation|**1**|No\Vasopressor|**1**|No\Rrt|**1**|No\Sepsis|**1**\Heart_rate|**2**\SPO2|**2**\Hemoglobin|**2**\BUN|**2**\

Glucose|**2**\Urine_output|**2**\Mannitol|**1**|No\Surgery|**1**|craniotomy\,

main_var = RPR_CAT|**1**|**1**\,

sub = Gender,

status = Expire_flag30,time = Time30, cp = **0**, cox_model = Gender2, output = **1**, rtf_a4 = **1**, dir = &dir.\Gender2-**30**天死亡亚组分析.rtf);

*== GCS;

**data** all_data;

set all_data;

if GCS < **14.0** then do;

gcs_new = **1**;

end;

else do;

gcs_new = **2**;

end;

**run**;

*Model 1;

%***ph_reg***(data = all_data, m_co_var = ,

main_var = RPR_CAT|**1**|**1**\,

sub = gcs_new,

status = Expire_flag30,time = Time30, cp = **0**, cox_model = gcs1, output = **1**, rtf_a4 = **1**, dir = &dir.\GCS1-**30**天死亡亚组分析.rtf);

*Model 2;

%***ph_reg***(data = all_data, m_co_var = Age|**2**\Ethnicity|**1**|Black\Admission_type|**1**|EMERGENCY\Ventilation|**1**|No\Vasopressor|**1**|No\Rrt|**1**|No\Sepsis|**1**\Heart_rate|**2**\SPO2|**2**\Hemoglobin|**2**\BUN|**2**\

Glucose|**2**\Urine_output|**2**\Mannitol|**1**|No\Surgery|**1**|craniotomy\,

main_var = RPR_CAT|**1**|**1**\,

sub = gcs_new,

status = Expire_flag30,time = Time30, cp = **0**, cox_model = gcs2, output = **1**, rtf_a4 = **1**, dir = &dir.\GCS2-**30**天死亡亚组分析.rtf);

*== Sofa（中位数）;

**data** all_data;

set all_data;

if Sofa < **3.0** then do;

Sofa_new = **1**;

end;

else do;

Sofa_new = **2**;

end;

**run**;

*Model 1;

%***ph_reg***(data = all_data, m_co_var = ,

main_var = RPR_CAT|**1**|**1**\,

sub = Sofa_new,

status = Expire_flag30,time = Time30, cp = **0**, cox_model = Sofa1, output = **1**, rtf_a4 = **1**, dir = &dir.\Sofa1-**30**天死亡亚组分析.rtf);

*Model 2;

%***ph_reg***(data = all_data, m_co_var = Age|**2**\Ethnicity|**1**|Black\Admission_type|**1**|EMERGENCY\Ventilation|**1**|No\Vasopressor|**1**|No\Rrt|**1**|No\Sepsis|**1**\Heart_rate|**2**\SPO2|**2**\Hemoglobin|**2**\BUN|**2**\

Glucose|**2**\Urine_output|**2**\Mannitol|**1**|No\Surgery|**1**|craniotomy\,

main_var = RPR_CAT|**1**|**1**\,

sub = Sofa_new,

status = Expire_flag30,time = Time30, cp = **0**, cox_model = Sofa2, output = **1**, rtf_a4 = **1**, dir = &dir.\Sofa2-**30**天死亡亚组分析.rtf);

*== Sapsii（中位数）;

**data** all_data;

set all_data;

if Sapsii < **33.0** then do;

Sapsii_new = **1**;

end;

else do;

Sapsii_new = **2**;

end;

**run**;

*Model 1;

%***ph_reg***(data = all_data, m_co_var = ,

main_var = RPR_CAT|**1**|**1**\,

sub = Sapsii_new,

status = Expire_flag30,time = Time30, cp = **0**, cox_model = Sapsii1, output = **1**, rtf_a4 = **1**, dir = &dir.\Sapsii1-**30**天死亡亚组分析.rtf);

*Model 2;

%***ph_reg***(data = all_data, m_co_var = Age|**2**\Ethnicity|**1**|Black\Admission_type|**1**|EMERGENCY\Ventilation|**1**|No\Vasopressor|**1**|No\Rrt|**1**|No\Sepsis|**1**\Heart_rate|**2**\SPO2|**2**\Hemoglobin|**2**\BUN|**2**\

Glucose|**2**\Urine_output|**2**\Mannitol|**1**|No\Surgery|**1**|craniotomy\,

main_var = RPR_CAT|**1**|**1**\,

sub = Sapsii_new,

status = Expire_flag30,time = Time30, cp = **0**, cox_model = Sapsii2, output = **1**, rtf_a4 = **1**, dir = &dir.\Sapsii2-**30**天死亡亚组分析.rtf);

*== Charlson_comorbidity_index（中位数）;

**data** all_data;

set all_data;

if Charlson_comorbidity_index < **4.0** then do;

Charlson_comorbidity_index_new = **1**;

end;

else do;

Charlson_comorbidity_index_new = **2**;

end;

**run**;

*Model 1;

%***ph_reg***(data = all_data, m_co_var = ,

main_var = RPR_CAT|**1**|**1**\,

sub = Charlson_comorbidity_index_new,

status = Expire_flag30,time = Time30, cp = **0**, cox_model = Charlson_comorbidity_index_new1, output = **1**, rtf_a4 = **1**, dir = &dir.\Charlson_comorbidity_index_new1-**30**天死亡亚组分析.rtf);

*Model 2;

%***ph_reg***(data = all_data, m_co_var = Age|**2**\Ethnicity|**1**|Black\Admission_type|**1**|EMERGENCY\Ventilation|**1**|No\Vasopressor|**1**|No\Rrt|**1**|No\Sepsis|**1**\Heart_rate|**2**\SPO2|**2**\Hemoglobin|**2**\BUN|**2**\

Glucose|**2**\Urine_output|**2**\Mannitol|**1**|No\Surgery|**1**|craniotomy\,

main_var = RPR_CAT|**1**|**1**\,

sub = Charlson_comorbidity_index_new,

status = Expire_flag30,time = Time30, cp = **0**, cox_model = Charlson_comorbidity_index_new2, output = **1**, rtf_a4 = **1**, dir = &dir.\Charlson_comorbidity_index_new2-**30**天死亡亚组分析.rtf);

*==Sepsis 脓毒症;

*Model 1;

%***ph_reg***(data = all_data, m_co_var = ,

main_var = RPR_CAT|**1**|**1**\,

sub = Sepsis,

status = Expire_flag30,time = Time30, cp = **0**, cox_model = Sepsis1, output = **1**, rtf_a4 = **1**, dir = &dir.\Sepsis1-**30**天死亡亚组分析.rtf);

*Model 2;

%***ph_reg***(data = all_data, m_co_var = Age|**2**\Ethnicity|**1**|Black\Admission_type|**1**|EMERGENCY\Ventilation|**1**|No\Vasopressor|**1**|No\Rrt|**1**|No\Heart_rate|**2**\SPO2|**2**\Hemoglobin|**2**\BUN|**2**\

Glucose|**2**\Urine_output|**2**\Mannitol|**1**|No\Surgery|**1**|craniotomy\,

main_var = RPR_CAT|**1**|**1**\,

sub = Sepsis,

status = Expire_flag30,time = Time30, cp = **0**, cox_model = Sepsis2, output = **1**, rtf_a4 = **1**, dir = &dir.\Sepsis2-**30**天死亡亚组分析.rtf);

*==Atrial_fibrillation 房颤;

*Model 1;

%***ph_reg***(data = all_data, m_co_var = ,

main_var = RPR_CAT|**1**|**1**\,

sub = Atrial_fibrillation,

status = Expire_flag30,time = Time30, cp = **0**, cox_model = Atrial_fibrillation1, output = **1**, rtf_a4 = **1**, dir = &dir.\Atrial_fibrillation1-**30**天死亡亚组分析.rtf);

*Model 2;

%***ph_reg***(data = all_data, m_co_var = Age|**2**\Ethnicity|**1**|Black\Admission_type|**1**|EMERGENCY\Ventilation|**1**|No\Vasopressor|**1**|No\Rrt|**1**|No\Sepsis|**1**\Heart_rate|**2**\SPO2|**2**\Hemoglobin|**2**\BUN|**2**\

Glucose|**2**\Urine_output|**2**\Mannitol|**1**|No\Surgery|**1**|craniotomy\,

main_var = RPR_CAT|**1**|**1**\,

sub = Atrial_fibrillation,

status = Expire_flag30,time = Time30, cp = **0**, cox_model = Atrial_fibrillation2, output = **1**, rtf_a4 = **1**, dir = &dir.\Atrial_fibrillation2-**30**天死亡亚组分析.rtf);

*==竞争风险模型;

**data** all_data;

set all_data;

if ICU_status = 'ICU_discharge' then ICU_status_new = **0**;

else if ICU_status = 'ICU_re_admission' then ICU_status_new = **1**;

else ICU_status_new = **2**;

if ICU_status = 'ICU_re_admission' then ICU_status_new1 = **1**;

else ICU_status_new1 = **0**;

**run**;

/*单因素*/

%***ph_reg***(data = all_data, m_co_var = ,

main_var = Age|**2**\Gender|**1**\Ethnicity|**1**\Insurance|**1**\Admission_type|**1**\First_careunit|**1**\Ventilation|**1**\Vasopressor|**1**\Rrt|**1**\Congestive_heart_failure|**1**\Sepsis|**1**\

Atrial_fibrillation|**1**\Hypertension|**1**\Malignant_cancer|**1**\Diabetes|**1**\Systolic|**2**\Diastolic|**2**\Respiratory_rate|**2**\Heart_rate|**2**\Temperature|**2**\SPO2|**2**\

Charlson_comorbidity_index|**2**\WBC|**2**\Hemoglobin|**2**\Hematocrit|**2**\Creatinine|**2**\INR|**2**\PT|**2**\BUN|**2**\Glucose|**2**\Bicarbonate|**2**\Sodium|**2**\Potassium|**2**\Chloride|**2**\Urine_output|**2**\

Mannitol|**1**\Anticoagulation|**1**\blood_transfusion|**1**\Surgery|**1**\neurodegeneration|**1**\RPR|**2**\RPR_CAT|**1**|**2**\,

status = ICU_status_new,time = ICU_follow_time, cp = **1**, cox_model = danyinsu, output = **1**, rtf_a4 = **0**, dir = &dir.\再入ICU竞争风险单因素.rtf);

/*多因素*/

%***ph_reg***(data = all_data, m_co_var = Gender|**1**\Ethnicity|**1**\Ventilation|**1**\Vasopressor|**1**\Sepsis|**1**\Systolic|**2**\Charlson_comorbidity_index|**2**\Hematocrit|**2**\PT|**2**\Glucose|**2**\Bicarbonate|**2**\

Mannitol|**1**\blood_transfusion|**1**\Surgery|**1**\neurodegeneration|**1**\RPR_CAT|**1**|**2**\,

main_var = ,

status = ICU_status_new,time = ICU_follow_time, cp = **1**, cox_model = danyinsu, output = **1**, rtf_a4 = **0**, dir = &dir.\再入ICU竞争风险多因素.rtf);

**proc** **freq** data = all_data;

table ICU_status_new Expire_flag30;

**run**;

# Data analysis and visualization：

####——————————————————————————————————————————————————1. 环境准备———————————————————————————————————————————####

##1.1 设置工作环境

library(dplyr)

library(plyr)

library(tableone)

library(MatchIt)

library(epiDisplay)

library(doBy)

library(survey)

library(rstudioapi) # 读取灵活路径

library(openxlsx) # 读取数据

library(ggplot2)

library(survival)

library(survminer) # 绘制km曲线

library(regplot)

library(rms) # 绘制列线图

library(timeROC) # 绘制ROC曲线

library(survivalROC)

library(ggthemes)

##1.2 设置工作路径

#install.packages("rstudioapi")#第一次跑需要安装这个包

dir <- dirname(rstudioapi::getActiveDocumentContext()$path)

setwd(dir)

##1.3 程序中使用的function(如果是临时写的function，简单注释功能)

#surv_roc_plot 可将多年份ROC输出到一张图里

#尽可能将用到的function复制过来便于程序复现，尽量避免使用source("")索引程序，如果function行数过多可以折叠起来

##1.4 读取数据

data1 = read.xlsx("Analysis_data.xlsx")

data1 %>% colnames #读取列名

data1 %>% dim #读取行列数

data1$RPR_CAT %>% table

data1$Expire_flag30 %>% table

data1$ICU_status %>% table

data1$ICU_status_new <- ifelse(data1$ICU_status == 'ICU_discharge', 0, ifelse(data1$ICU_status == 'ICU_re_admission', 1, ifelse(data1$ICU_status == 'ICU_death', 2, 3)))

data1$ICU_status_new %>% table()

data1 %>% str

train_data = read.csv('train_dataset.csv')

train_data$ICU_status_new <- ifelse(train_data$ICU_status == 'ICU_discharge', 0, ifelse(train_data$ICU_status == 'ICU_re_admission', 1, ifelse(train_data$ICU_status == 'ICU_death', 2, 3)))

train_data$ICU_status_new %>% table()

test_data = read.csv('test_dataset.csv')

test_data$ICU_status_new <- ifelse(test_data$ICU_status == 'ICU_discharge', 0, ifelse(test_data$ICU_status == 'ICU_re_admission', 1, ifelse(test_data$ICU_status == 'ICU_death', 2, 3)))

test_data$ICU_status_new %>% table()

####——————————————————————————————————————————————————2. 数据整理———————————————————————————————————————————####

## 数据清洗过程已经在jupyter notebook中完成一部分

##2.1 转换数据类型，处理数据结构

library(magrittr)

data1 %<>% dplyr::mutate_at(c('Gender', 'Ethnicity', 'Insurance', 'First_careunit', 'Admission_type', 'Congestive_heart_failure', 'Sepsis', 'Atrial_fibrillation', 'Hypertension', 'Malignant_cancer', 'Ventilation', 'Vasopressor', 'Rrt', 'Mannitol', 'Surgery', 'Anticoagulation', 'Diabetes', 'blood_transfusion', 'Diabetes', 'neurodegeneration'),~as.factor(.x))

train_data %<>% dplyr::mutate_at(c('Gender', 'Ethnicity', 'Insurance', 'First_careunit', 'Admission_type', 'Congestive_heart_failure', 'Sepsis', 'Atrial_fibrillation', 'Hypertension', 'Malignant_cancer', 'Ventilation', 'Vasopressor', 'Rrt', 'Mannitol', 'Surgery', 'Anticoagulation', 'Diabetes', 'blood_transfusion', 'Diabetes', 'neurodegeneration'),~as.factor(.x))

test_data %<>% dplyr::mutate_at(c('Gender', 'Ethnicity', 'Insurance', 'First_careunit', 'Admission_type', 'Congestive_heart_failure', 'Sepsis', 'Atrial_fibrillation', 'Hypertension', 'Malignant_cancer', 'Ventilation', 'Vasopressor', 'Rrt', 'Mannitol', 'Surgery', 'Anticoagulation', 'Diabetes', 'blood_transfusion', 'Diabetes', 'neurodegeneration'),~as.factor(.x))

data1$RPR_CAT <- factor(data1$RPR_CAT, levels = c(1,2,3))

train_data$RPR_CAT <- factor(train_data$RPR_CAT, levels = c(1,2,3))

test_data$RPR_CAT <- factor(test_data$RPR_CAT, levels = c(1,2,3))

####———————————————————————————————————————————————————3. 输出图———————————————————————————————————————————####

## 3.1 km曲线

km1<- survfit(Surv(Time30, Expire_flag30)~ RPR_CAT, data = data1)

pairwise_survdiff(Surv(Time30,Expire_flag30) ~ RPR_CAT,data1,p.adjust.method = "BH")

# Expire_flag30

pdf("30天死亡km曲线.pdf", width = 10, height = 8)

ggsurvplot(km1,

data = data1, censor=F, #去掉加号

pval = T,#P值是否显示

conf.int=F,#可信区间绘制

pval.method = T,#P值的方法是否显示

palette = "lancet",

#p值及其方法显示的坐标

pval.method.coord=c(0,0.4),

pval.coord=c(0,0.2),

surv.median.line = "none",#中位生存是否标识，无："none", 横线加垂线："hv", 横线："h", 垂线："v"

legend = c(0.85, 0.25),#图例位置

# 图例标题、图例标签

legend.title='RPR',

break.time.by = 5, #x轴间隔多长给个标签

legend.labs = c("<=Q1", "Q1 to Q2", ">Q2"),

# ggtheme = theme_light(), # 网格

risk.table = "abs_pct",

risk.table.height = 0.3,

risk.table.y.text = F,

xlab="Time (Days)",

ylab="Probability of Survival",

title = "Probability of Survival for 30-days",

newpage = FALSE)

dev.off()

## 3.2 RCS曲线

dd <- datadist(data1)

options(datadist = "dd")

S <- Surv(data1$Time30, data1$Expire_flag30 == 1)

fit <- cph(S ~ rcs(RPR, 3), x = TRUE, y = TRUE, data = data1)

anova(fit)

Pre0 <- Predict(fit, RPR, fun = exp, type = "predictions", ref.zero = TRUE, conf.int = 0.95, digits = 2)

Pre0$yhat_1 <- abs(Pre0$yhat-1)

pdf("RCS曲线.pdf", width = 10, height = 8)

ggplot()+

geom_line(data=Pre0, #数据来源

aes(RPR,yhat),#xy轴的数据

linetype="solid",#曲线加粗

size=1,

alpha=0.9,

colour="orange")+

geom_ribbon(data=Pre0, #加入置信区间

aes(RPR,

ymin=lower,

ymax=upper),

alpha=0.3,

fill="orange")+

theme_classic()+

geom_vline(xintercept = Pre0[which(Pre0$yhat_1 == min(Pre0$yhat_1)), ]$RPR, linetype = 2, size = 0.70) +

annotate("text", x=Pre0[which(Pre0$yhat_1 == min(Pre0$yhat_1)), ]$RPR, y=0, parse = TRUE, label = Pre0[which(Pre0$yhat_1 == min(Pre0$yhat_1)), ]$RPR) +

geom_hline(yintercept=1,linetype=2,size=0.70)+ #y=1的水平线

labs(title="RCS Curve of 30-days death risk with RPR",

x="RPR",

y="HR (95%CI)"

) +

theme(axis.title.x = element_text(size = 12), axis.title.y = element_text(size = 12)) +

theme_par()

dev.off()

## 3.2 ROC曲线

library(pec)

# source("statistics_matrix.R")

# RPR_CAT

model1 <- coxph(Surv(Time30, Expire_flag30) ~ RPR_CAT + Age + Ethnicity + First_careunit + Ventilation + Vasopressor + Rrt + Sepsis + Heart_rate + SPO2 + Hemoglobin + BUN + Glucose + Urine_output + Mannitol + Anticoagulation + Surgery + neurodegeneration, data = train_data)

summary(model1)$concordance

model1 <- cph(Surv(Time30, Expire_flag30) ~ RPR_CAT + Age + Ethnicity + First_careunit + Ventilation + Vasopressor + Rrt + Sepsis + Heart_rate + SPO2 + Hemoglobin + BUN + Glucose + Urine_output + Mannitol + Anticoagulation + Surgery + neurodegeneration, data = train_data, surv = T)

# 训练集概率

prob_rpr_train <- 1-predictSurvProb(model1, newdata = train_data,times = c(15, 30))

train_data$rpr_15_train <- prob_rpr_train[,1]

train_data$rpr_30_train <- prob_rpr_train[,2]

# 测试集概率

prob_rpr_test <- 1-predictSurvProb(model1, newdata = test_data,times = c(15, 30))

test_data$rpr_15_test <- prob_rpr_test[,1]

test_data$rpr_30_test <- prob_rpr_test[,2]

# sm1_15 = statistics_matrix(data1$Expire_flag15,data1$prob1_15,cutoff='auto')

# sm1_30 = statistics_matrix(data1$Expire_flag30,data1$prob1_30,cutoff='auto')

# Sofa

model2 <- coxph(Surv(Time30, Expire_flag30) ~ Sofa, data = train_data)

summary(model2)$concordance

model2 <- cph(Surv(Time30, Expire_flag30) ~ Sofa, data = train_data, surv = T)

# 训练集概率

prob_sofa_train <- 1-predictSurvProb(model2, newdata = train_data, times = c(15, 30))

train_data$sofa_15_train <- prob_sofa_train[,1]

train_data$sofa_30_train <- prob_sofa_train[,2]

# 测试集概率

prob_sofa_test <- 1-predictSurvProb(model2, newdata = test_data, times = c(15, 30))

test_data$sofa_15_test <- prob_sofa_test[,1]

test_data$sofa_30_test <- prob_sofa_test[,2]

# sm2 = statistics_matrix(data1$Expire_flag30, data1$prob2, cutoff='auto')

# Sapsii

model3 <- coxph(Surv(Time30, Expire_flag30) ~ Sapsii, data = train_data)

summary(model3)$concordance

model3 <- cph(Surv(Time30, Expire_flag30) ~ Sapsii, data = train_data, surv = T)

# 训练集概率

prob_sapsii_train <- 1- predictSurvProb(model3, newdata = train_data,times = c(15, 30))

train_data$sapsii_15_train <- prob_sapsii_train[,1]

train_data$sapsii_30_train <- prob_sapsii_train[,2]

# 测试集概率

prob_sapsii_test <- 1- predictSurvProb(model3, newdata = test_data,times = c(15, 30))

test_data$sapsii_15_test <- prob_sapsii_test[,1]

test_data$sapsii_30_test <- prob_sapsii_test[,2]

# sm3 = statistics_matrix(data1$Expire_flag30, data1$prob3, cutoff='auto')

# qsofa

model4 <- coxph(Surv(Time30, Expire_flag30) ~ qsofa, data = train_data)

summary(model4)$concordance

model4 <- cph(Surv(Time30, Expire_flag30) ~ qsofa, data = train_data, surv = T)

# 训练集概率

prob_qsofa_train <- 1- predictSurvProb(model4, newdata = train_data, times = c(15, 30))

train_data$qsofa_15_train <- prob_qsofa_train[,1]

train_data$qsofa_30_train <- prob_qsofa_train[,2]

# 测试集概率

prob_qsofa_test <- 1- predictSurvProb(model4, newdata = test_data, times = c(15, 30))

test_data$qsofa_15_test <- prob_qsofa_test[,1]

test_data$qsofa_30_test <- prob_qsofa_test[,2]

# sm3 = statistics_matrix(data1$Expire_flag30, data1$prob3, cutoff='auto')

# 输出data1在python上完成roc曲线绘制

write.csv(train_data, "data_pred_train.csv")

write.csv(test_data, "data_pred_test.csv")

# 读取R文件输出的数据集data_pred_train, data_pred_test

data_pred_train = pd.read_csv("data_pred_train.csv")

train_cols = ['rpr_15_train', 'rpr_30_train', 'sofa_15_train', 'sofa_30_train', 'sapsii_15_train', 'sapsii_30_train', 'qsofa_15_train', 'qsofa_30_train', 'Expire_flag15', 'Expire_flag30']

data_pred_train = data_pred_train[train_cols]

data_pred_test = pd.read_csv("data_pred_test.csv")

test_cols = ['rpr_15_test', 'rpr_30_test', 'sofa_15_test', 'sofa_30_test', 'sapsii_15_test', 'sapsii_30_test', 'qsofa_15_test', 'qsofa_30_test', 'Expire_flag15', 'Expire_flag30']

data_pred_test = data_pred_test[test_cols]

data_pred_train

print(data_pred_train.columns.tolist())

# RPR

# 训练集

pred_rpr_15_train = metrics1_v2.model_parameter(data_pred_train.Expire_flag15, data_pred_train.rpr_15_train, auc_ci_type = 1,curve_show = True)

# 测试集

pred_rpr_15_test = metrics1_v2.model_parameter(data_pred_test.Expire_flag15, data_pred_test.rpr_15_test, auc_ci_type = 1,curve_show = True, cut_off = float(pred_rpr_15_train.Cut_off.values))

pred_rpr_15 = pd.concat([pred_rpr_15_train, pred_rpr_15_test])

pred_rpr_15

# 训练集

pred_rpr_30_train = metrics1_v2.model_parameter(data_pred_train.Expire_flag30, data_pred_train.rpr_30_train, auc_ci_type = 1,curve_show = True)

# 测试集

pred_rpr_30_test = metrics1_v2.model_parameter(data_pred_test.Expire_flag30, data_pred_test.rpr_30_test, auc_ci_type = 1,curve_show = True, cut_off = float(pred_rpr_30_train.Cut_off.values))

pred_rpr_30 = pd.concat([pred_rpr_30_train, pred_rpr_30_test])

pred_rpr_30

# Sofa

# 训练集

pred_sofa_15_train = metrics1_v2.model_parameter(data_pred_train.Expire_flag15, data_pred_train.sofa_15_train, auc_ci_type = 1,curve_show = True)

# 测试集

pred_sofa_15_test = metrics1_v2.model_parameter(data_pred_test.Expire_flag15, data_pred_test.sofa_15_test, auc_ci_type = 1,curve_show = True, cut_off = float(pred_sofa_15_train.Cut_off.values))

pred_sofa_15 = pd.concat([pred_sofa_15_train, pred_sofa_15_test])

pred_sofa_15

# 训练集

pred_sofa_30_train = metrics1_v2.model_parameter(data_pred_train.Expire_flag30, data_pred_train.sofa_30_train, auc_ci_type = 1,curve_show = True)

# 测试集

pred_sofa_30_test = metrics1_v2.model_parameter(data_pred_test.Expire_flag30, data_pred_test.sofa_30_test, auc_ci_type = 1,curve_show = True, cut_off = float(pred_sofa_30_train.Cut_off.values))

pred_sofa_30 = pd.concat([pred_sofa_30_train, pred_sofa_30_test])

pred_sofa_30

# Sapsii

# 训练集

pred_sapsii_15_train = metrics1_v2.model_parameter(data_pred_train.Expire_flag15, data_pred_train.sapsii_15_train, auc_ci_type = 1,curve_show = True)

# 测试集

pred_sapsii_15_test = metrics1_v2.model_parameter(data_pred_test.Expire_flag15, data_pred_test.sapsii_15_test, auc_ci_type = 1,curve_show = True, cut_off = float(pred_sapsii_15_train.Cut_off.values))

pred_sapsii_15 = pd.concat([pred_sapsii_15_train, pred_sapsii_15_test])

pred_sapsii_15

# 训练集

pred_sapsii_30_train = metrics1_v2.model_parameter(data_pred_train.Expire_flag30, data_pred_train.sapsii_30_train, auc_ci_type = 1,curve_show = True)

# 测试集

pred_sapsii_30_test = metrics1_v2.model_parameter(data_pred_test.Expire_flag30, data_pred_test.sapsii_30_test, auc_ci_type = 1,curve_show = True, cut_off = float(pred_sapsii_30_train.Cut_off.values))

pred_sapsii_30 = pd.concat([pred_sapsii_30_train, pred_sapsii_30_test])

pred_sapsii_30

# qsofa

# 训练集

pred_qsofa_15_train = metrics1_v2.model_parameter(data_pred_train.Expire_flag15, data_pred_train.qsofa_15_train, auc_ci_type = 1,curve_show = True)

# 测试集

pred_qsofa_15_test = metrics1_v2.model_parameter(data_pred_test.Expire_flag15, data_pred_test.qsofa_15_test, auc_ci_type = 1,curve_show = True, cut_off = float(pred_qsofa_15_train.Cut_off.values))

pred_qsofa_15 = pd.concat([pred_qsofa_15_train, pred_qsofa_15_test])

pred_qsofa_15

# 训练集

pred_qsofa_30_train = metrics1_v2.model_parameter(data_pred_train.Expire_flag30, data_pred_train.qsofa_30_train, auc_ci_type = 1,curve_show = True)

# 测试集

pred_qsofa_30_test = metrics1_v2.model_parameter(data_pred_test.Expire_flag30, data_pred_test.qsofa_30_test, auc_ci_type = 1,curve_show = True, cut_off = float(pred_qsofa_30_train.Cut_off.values))

pred_qsofa_30 = pd.concat([pred_qsofa_30_train, pred_qsofa_30_test])

pred_qsofa_30

## 绘制整体ROC曲线

#计算真正率和假正率

fpr,tpr,threshold = roc_curve(data_pred_train.Expire_flag15,data_pred_train.rpr_15_train) # 前面放真值，就是真正的结果值，后面就是预测的概率值；

fpr2,tpr2,threshold2 = roc_curve(data_pred_train.Expire_flag15,data_pred_train.sofa_15_train)

fpr3,tpr3,threshold3 = roc_curve(data_pred_train.Expire_flag15,data_pred_train.sapsii_15_train)

fpr4,tpr4,threshold4 = roc_curve(data_pred_train.Expire_flag15,data_pred_train.qsofa_15_train)

fpr5,tpr5,threshold5 = roc_curve(data_pred_train.Expire_flag30,data_pred_train.rpr_30_train) # 前面放真值，就是真正的结果值，后面就是预测的概率值；

fpr6,tpr6,threshold6 = roc_curve(data_pred_train.Expire_flag30,data_pred_train.sofa_30_train)

fpr7,tpr7,threshold7 = roc_curve(data_pred_train.Expire_flag30,data_pred_train.sapsii_15_train)

fpr8,tpr8,threshold8 = roc_curve(data_pred_train.Expire_flag30,data_pred_train.qsofa_30_train)

# 画图

plt.figure(figsize = (15,7.5),facecolor='white')

lw = 2

plt.subplot(1,2,1)

plt.plot(fpr, tpr, color='darkorange',

lw=lw, label='RPR AUC (95%CI) = {}'.format(pred_rpr_15_train.loc[:,'AUC(95%CI)'][0])) ###假正率为横坐标，真正率为纵坐标做曲线

plt.plot(fpr2, tpr2, color='darkcyan',

lw=lw, label='Sofa AUC (95%CI) = {}'.format(pred_sofa_15_train.loc[:,'AUC(95%CI)'][0]))

plt.plot(fpr3, tpr3, color='SkyBlue',

lw=lw, label='Sapsii AUC (95%CI) = {}'.format(pred_sapsii_15_train.loc[:,'AUC(95%CI)'][0])) ###假正率为横坐标，真正率为纵坐标做曲线

plt.plot(fpr4, tpr4, color='Salmon',

lw=lw, label='qsofa AUC (95%CI) = {}'.format(pred_qsofa_15_train.loc[:,'AUC(95%CI)'][0]))

plt.plot([0, 1], [0, 1], color='navy', lw=lw, linestyle='--')

plt.xlim([0.0, 1.0])

plt.ylim([0.0, 1.05])

plt.xlabel('1-Specificity',fontdict = {'fontsize':18},labelpad = 15)

plt.ylabel('Sensitivity',fontdict = {'fontsize':18},labelpad = 15)

plt.title('ROC Curve 15 Days Survival in Train Dataset',fontdict={"fontsize":20},pad = 15)

plt.legend(loc="lower right",fontsize = 13)

plt.tick_params(labelsize = 18)

plt.subplot(1,2,2)

plt.plot(fpr5, tpr5, color='darkorange',

lw=lw, label='RPR AUC (95%CI) = {}'.format(pred_rpr_30_train.loc[:,'AUC(95%CI)'][0])) ###假正率为横坐标，真正率为纵坐标做曲线

plt.plot(fpr6, tpr6, color='darkcyan',

lw=lw, label='Sofa AUC (95%CI) = {}'.format(pred_sofa_30_train.loc[:,'AUC(95%CI)'][0]))

plt.plot(fpr7, tpr7, color='SkyBlue',

lw=lw, label='Sapsii AUC (95%CI) = {}'.format(pred_sapsii_30_train.loc[:,'AUC(95%CI)'][0])) ###假正率为横坐标，真正率为纵坐标做曲线

plt.plot(fpr8, tpr8, color='Salmon',

lw=lw, label='qsofa AUC (95%CI) = {}'.format(pred_qsofa_30_train.loc[:,'AUC(95%CI)'][0]))

plt.plot([0, 1], [0, 1], color='navy', lw=lw, linestyle='--')

plt.xlim([0.0, 1.0])

plt.ylim([0.0, 1.05])

plt.xlabel('1-Specificity',fontdict = {'fontsize':18},labelpad = 15)

plt.ylabel('Sensitivity',fontdict = {'fontsize':18},labelpad = 15)

plt.title('ROC Curve 30 Days Survival in Train Dataset',fontdict={"fontsize":20},pad = 15)

plt.legend(loc="lower right",fontsize = 13)

plt.tick_params(labelsize = 18)

plt.savefig('ROC-训练集.pdf',dpi = 300, bbox_inches = 'tight')

plt.show()

## 绘制整体ROC曲线

#计算真正率和假正率

fpr,tpr,threshold = roc_curve(data_pred_test.Expire_flag15,data_pred_test.rpr_15_test) # 前面放真值，就是真正的结果值，后面就是预测的概率值；

fpr2,tpr2,threshold2 = roc_curve(data_pred_test.Expire_flag15,data_pred_test.sofa_15_test)

fpr3,tpr3,threshold3 = roc_curve(data_pred_test.Expire_flag15,data_pred_test.sapsii_15_test)

fpr4,tpr4,threshold4 = roc_curve(data_pred_test.Expire_flag15,data_pred_test.qsofa_15_test)

fpr5,tpr5,threshold5 = roc_curve(data_pred_test.Expire_flag30,data_pred_test.rpr_30_test) # 前面放真值，就是真正的结果值，后面就是预测的概率值；

fpr6,tpr6,threshold6 = roc_curve(data_pred_test.Expire_flag30,data_pred_test.sofa_30_test)

fpr7,tpr7,threshold7 = roc_curve(data_pred_test.Expire_flag30,data_pred_test.sapsii_30_test)

fpr8,tpr8,threshold8 = roc_curve(data_pred_test.Expire_flag30,data_pred_test.qsofa_30_test)

# 画图

plt.figure(figsize = (15,7.5),facecolor='white')

lw = 2

plt.subplot(1,2,1)

plt.plot(fpr, tpr, color='darkorange',

lw=lw, label='RPR AUC (95%CI) = {}'.format(pred_rpr_15_test.loc[:,'AUC(95%CI)'][0])) ###假正率为横坐标，真正率为纵坐标做曲线

plt.plot(fpr2, tpr2, color='darkcyan',

lw=lw, label='Sofa AUC (95%CI) = {}'.format(pred_sofa_15_test.loc[:,'AUC(95%CI)'][0]))

plt.plot(fpr3, tpr3, color='SkyBlue',

lw=lw, label='Sapsii AUC (95%CI) = {}'.format(pred_sapsii_15_test.loc[:,'AUC(95%CI)'][0])) ###假正率为横坐标，真正率为纵坐标做曲线

plt.plot(fpr4, tpr4, color='Salmon',

lw=lw, label='qsofa AUC (95%CI) = {}'.format(pred_qsofa_15_test.loc[:,'AUC(95%CI)'][0]))

plt.plot([0, 1], [0, 1], color='navy', lw=lw, linestyle='--')

plt.xlim([0.0, 1.0])

plt.ylim([0.0, 1.05])

plt.xlabel('1-Specificity',fontdict = {'fontsize':18},labelpad = 15)

plt.ylabel('Sensitivity',fontdict = {'fontsize':18},labelpad = 15)

plt.title('ROC Curve 15 Days Survival in Test Dataset',fontdict={"fontsize":20},pad = 15)

plt.legend(loc="lower right",fontsize = 13)

plt.tick_params(labelsize = 18)

plt.subplot(1,2,2)

plt.plot(fpr5, tpr5, color='darkorange',

lw=lw, label='RPR AUC (95%CI) = {}'.format(pred_rpr_30_test.loc[:,'AUC(95%CI)'][0])) ###假正率为横坐标，真正率为纵坐标做曲线

plt.plot(fpr6, tpr6, color='darkcyan',

lw=lw, label='Sofa AUC (95%CI) = {}'.format(pred_sofa_30_test.loc[:,'AUC(95%CI)'][0]))

plt.plot(fpr7, tpr7, color='SkyBlue',

lw=lw, label='Sapsii AUC (95%CI) = {}'.format(pred_sapsii_30_test.loc[:,'AUC(95%CI)'][0])) ###假正率为横坐标，真正率为纵坐标做曲线

plt.plot(fpr8, tpr8, color='Salmon',

lw=lw, label='qsofa AUC (95%CI) = {}'.format(pred_qsofa_30_test.loc[:,'AUC(95%CI)'][0]))

plt.plot([0, 1], [0, 1], color='navy', lw=lw, linestyle='--')

plt.xlim([0.0, 1.0])

plt.ylim([0.0, 1.05])

plt.xlabel('1-Specificity',fontdict = {'fontsize':18},labelpad = 15)

plt.ylabel('Sensitivity',fontdict = {'fontsize':18},labelpad = 15)

plt.title('ROC Curve 30 Days Survival in Test Dataset',fontdict={"fontsize":20},pad = 15)

plt.legend(loc="lower right",fontsize = 13)

plt.tick_params(labelsize = 18)

plt.savefig('ROC-测试集.pdf',dpi = 300, bbox_inches = 'tight')

plt.show()

# 校准曲线

#校准曲线--分类变量

plt.figure(figsize=(16, 10),facecolor='white')

grid = plt.GridSpec(3, 2, wspace=0.3, hspace=0.5)

plt.subplot(grid[0:2, 0])

plt.title("15 Days Survival Calibration Curve in Train Dataset",fontdict={'fontsize':20},pad = 15)

plt.plot([0, 1], [0, 1], linestyle='--', color='navy',lw=2,label="Perfectly calibrated")

fraction_of_positives,mean_predicted_value = calibration_curve(data_pred_train.Expire_flag15,data_pred_train.rpr_15_train, n_bins=4)

fraction_of_positives2,mean_predicted_value2 = calibration_curve(data_pred_train.Expire_flag15,data_pred_train.sofa_15_train, n_bins=4)

fraction_of_positives3,mean_predicted_value3 = calibration_curve(data_pred_train.Expire_flag15,data_pred_train.sapsii_15_train, n_bins=4)# 这里修改，n_bins修改多少个点

fraction_of_positives4,mean_predicted_value4 = calibration_curve(data_pred_train.Expire_flag15,data_pred_train.qsofa_15_train, n_bins=4)

plt.plot(mean_predicted_value, fraction_of_positives, color='darkorange',lw=2,label = 'RPR')

plt.plot(mean_predicted_value2, fraction_of_positives2, color='darkcyan',lw=2,label = 'Sofa')

plt.plot(mean_predicted_value3, fraction_of_positives3, color='SkyBlue',lw=2,label = 'Sapsii')

plt.plot(mean_predicted_value4, fraction_of_positives4, color='Salmon',lw=2,label = 'qsofa')

plt.legend(fontsize=15)

plt.ylabel("Actual values",fontsize=15)

plt.xlabel("Predicted values",fontsize=15)

plt.xticks(fontsize=12)

plt.yticks(fontsize=12)

plt.ylim([0, 1.05])

plt.subplot(grid[2, 0])

plt.hist(data_pred_train.rpr_15_train, range=(0, 1), bins=10, histtype="step", lw=2,color = 'darkorange',label='RPR')

plt.hist(data_pred_train.sofa_15_train, range=(0, 1), bins=10, histtype="step", lw=2,color = 'darkcyan',label='Sofa')

plt.hist(data_pred_train.sapsii_15_train, range=(0, 1), bins=10, histtype="step", lw=2,color = 'SkyBlue',label='Sapsii')

plt.hist(data_pred_train.qsofa_15_train, range=(0, 1), bins=10, histtype="step", lw=2,color = 'Salmon',label='qsofa')

plt.xlabel("Mean predicted value",fontsize=15)

plt.ylabel("Count",fontsize=15)

plt.xticks(fontsize=12)

plt.yticks(fontsize=12)

plt.legend(fontsize = 15,loc='upper right')

plt.subplot(grid[0:2, 1])

plt.title("30 Days Survival Calibration Curve in Train Dataset",fontdict={'fontsize':20},pad = 15)

plt.plot([0, 1], [0, 1], linestyle='--', color='navy',lw=2,label="Perfectly calibrated")

fraction_of_positives5,mean_predicted_value5 = calibration_curve(data_pred_train.Expire_flag30,data_pred_train.rpr_30_train, n_bins=4)

fraction_of_positives6,mean_predicted_value6 = calibration_curve(data_pred_train.Expire_flag30,data_pred_train.sofa_30_train, n_bins=4)

fraction_of_positives7,mean_predicted_value7 = calibration_curve(data_pred_train.Expire_flag30,data_pred_train.sapsii_30_train, n_bins=4)# 这里修改，n_bins修改多少个点

fraction_of_positives8,mean_predicted_value8 = calibration_curve(data_pred_train.Expire_flag30,data_pred_train.qsofa_30_train, n_bins=4)

plt.plot(mean_predicted_value5, fraction_of_positives5, color='darkorange',lw=2,label = 'RPR')

plt.plot(mean_predicted_value6, fraction_of_positives6, color='darkcyan',lw=2,label = 'Sofa')

plt.plot(mean_predicted_value7, fraction_of_positives7, color='SkyBlue',lw=2,label = 'Sapsii')

plt.plot(mean_predicted_value8, fraction_of_positives8, color='Salmon',lw=2,label = 'qsofa')

plt.legend(fontsize=15)

plt.ylabel("Actual values",fontsize=15)

plt.xlabel("Predicted values",fontsize=15)

plt.xticks(fontsize=12)

plt.yticks(fontsize=12)

plt.ylim([0, 1.05])

plt.subplot(grid[2, 1])

plt.hist(data_pred_train.rpr_30_train, range=(0, 1), bins=10, histtype="step", lw=2,color = 'darkorange',label='RPR')

plt.hist(data_pred_train.sofa_30_train, range=(0, 1), bins=10, histtype="step", lw=2,color = 'darkcyan',label='Sofa')

plt.hist(data_pred_train.sapsii_30_train, range=(0, 1), bins=10, histtype="step", lw=2,color = 'SkyBlue',label='Sapsii')

plt.hist(data_pred_train.qsofa_30_train, range=(0, 1), bins=10, histtype="step", lw=2,color = 'Salmon',label='qsofa')

plt.xlabel("Mean predicted value",fontsize=15)

plt.ylabel("Count",fontsize=15)

plt.xticks(fontsize=12)

plt.yticks(fontsize=12)

plt.legend(fontsize = 15,loc='upper right')

plt.savefig('Calibration_Curve-训练集.pdf',dpi = 300, bbox_inches = 'tight')

plt.show()

# 校准曲线

#校准曲线--分类变量

plt.figure(figsize=(16, 10),facecolor='white')

grid = plt.GridSpec(3, 2, wspace=0.3, hspace=0.5)

plt.subplot(grid[0:2, 0])

plt.title("15 Days Survival Calibration Curve in Test Dataset",fontdict={'fontsize':20},pad = 15)

plt.plot([0, 1], [0, 1], linestyle='--', color='navy',lw=2,label="Perfectly calibrated")

fraction_of_positives,mean_predicted_value = calibration_curve(data_pred_test.Expire_flag15,data_pred_test.rpr_15_test, n_bins=4)

fraction_of_positives2,mean_predicted_value2 = calibration_curve(data_pred_test.Expire_flag15,data_pred_test.sofa_15_test, n_bins=4)

fraction_of_positives3,mean_predicted_value3 = calibration_curve(data_pred_test.Expire_flag15,data_pred_test.sapsii_15_test, n_bins=4)# 这里修改，n_bins修改多少个点

fraction_of_positives4,mean_predicted_value4 = calibration_curve(data_pred_test.Expire_flag15,data_pred_test.qsofa_15_test, n_bins=4)

plt.plot(mean_predicted_value, fraction_of_positives, color='darkorange',lw=2,label = 'RPR')

plt.plot(mean_predicted_value2, fraction_of_positives2, color='darkcyan',lw=2,label = 'Sofa')

plt.plot(mean_predicted_value3, fraction_of_positives3, color='SkyBlue',lw=2,label = 'Sapsii')

plt.plot(mean_predicted_value4, fraction_of_positives4, color='Salmon',lw=2,label = 'qsofa')

plt.legend(fontsize=15)

plt.ylabel("Actual values",fontsize=15)

plt.xlabel("Predicted values",fontsize=15)

plt.xticks(fontsize=12)

plt.yticks(fontsize=12)

plt.ylim([0, 1.05])

plt.subplot(grid[2, 0])

plt.hist(data_pred_test.rpr_15_test, range=(0, 1), bins=10, histtype="step", lw=2,color = 'darkorange',label='RPR')

plt.hist(data_pred_test.sofa_15_test, range=(0, 1), bins=10, histtype="step", lw=2,color = 'darkcyan',label='Sofa')

plt.hist(data_pred_test.sapsii_15_test, range=(0, 1), bins=10, histtype="step", lw=2,color = 'SkyBlue',label='Sapsii')

plt.hist(data_pred_test.qsofa_15_test, range=(0, 1), bins=10, histtype="step", lw=2,color = 'Salmon',label='qsofa')

plt.xlabel("Mean predicted value",fontsize=15)

plt.ylabel("Count",fontsize=15)

plt.xticks(fontsize=12)

plt.yticks(fontsize=12)

plt.legend(fontsize = 15,loc='upper right')

plt.subplot(grid[0:2, 1])

plt.title("30 Days Survival Calibration Curve in Test Dataset",fontdict={'fontsize':20},pad = 15)

plt.plot([0, 1], [0, 1], linestyle='--', color='navy',lw=2,label="Perfectly calibrated")

fraction_of_positives5,mean_predicted_value5 = calibration_curve(data_pred_test.Expire_flag30,data_pred_test.rpr_30_test, n_bins=4)

fraction_of_positives6,mean_predicted_value6 = calibration_curve(data_pred_test.Expire_flag30,data_pred_test.sofa_30_test, n_bins=4)

fraction_of_positives7,mean_predicted_value7 = calibration_curve(data_pred_test.Expire_flag30,data_pred_test.sapsii_30_test, n_bins=4)# 这里修改，n_bins修改多少个点

fraction_of_positives8,mean_predicted_value8 = calibration_curve(data_pred_test.Expire_flag30,data_pred_test.qsofa_30_test, n_bins=4)

plt.plot(mean_predicted_value5, fraction_of_positives5, color='darkorange',lw=2,label = 'RPR')

plt.plot(mean_predicted_value6, fraction_of_positives6, color='darkcyan',lw=2,label = 'Sofa')

plt.plot(mean_predicted_value7, fraction_of_positives7, color='SkyBlue',lw=2,label = 'Sapsii')

plt.plot(mean_predicted_value8, fraction_of_positives8, color='Salmon',lw=2,label = 'qsofa')

plt.legend(fontsize=15)

plt.ylabel("Actual values",fontsize=15)

plt.xlabel("Predicted values",fontsize=15)

plt.xticks(fontsize=12)

plt.yticks(fontsize=12)

plt.ylim([0, 1.05])

plt.subplot(grid[2, 1])

plt.hist(data_pred_test.rpr_30_test, range=(0, 1), bins=10, histtype="step", lw=2,color = 'darkorange',label='RPR')

plt.hist(data_pred_test.sofa_30_test, range=(0, 1), bins=10, histtype="step", lw=2,color = 'darkcyan',label='Sofa')

plt.hist(data_pred_test.sapsii_30_test, range=(0, 1), bins=10, histtype="step", lw=2,color = 'SkyBlue',label='Sapsii')

plt.hist(data_pred_test.qsofa_30_test, range=(0, 1), bins=10, histtype="step", lw=2,color = 'Salmon',label='qsofa')

plt.xlabel("Mean predicted value",fontsize=15)

plt.ylabel("Count",fontsize=15)

plt.xticks(fontsize=12)

plt.yticks(fontsize=12)

plt.legend(fontsize = 15,loc='upper right')

plt.savefig('Calibration_Curve-测试集.pdf',dpi = 300, bbox_inches = 'tight')

plt.show()

pred_rpr_15["Dataset"] = "RPR15"

pred_sofa_15["Dataset"] = "Sofa15"

pred_sapsii_15["Dataset"] = "Sapsii15"

pred_qsofa_15["Dataset"] = "qsofa15"

pred_rpr_30["Dataset"] = "RPR30"

pred_sofa_30["Dataset"] = "Sofa30"

pred_sapsii_30["Dataset"] = "Sapsii30"

pred_qsofa_30["Dataset"] = "qsofa30"

predict_matrix = pd.concat([pred_rpr_15, pred_sofa_15, pred_sapsii_15, pred_qsofa_15, pred_rpr_30, pred_sofa_30,

pred_sapsii_30, pred_qsofa_30], axis = 0)

predict_matrix['Train_test'] = np.tile(['训练集', '测试集'], 8)

predict_matrix = predict_matrix.set_index(['Dataset', 'Train_test'])

predict_matrix

predict_matrix.to_csv('predict_matrix.csv')

import numpy as np

from matplotlib import pyplot as plt

import scipy.stats as st

from sklearn import metrics

class DelongTest():

def __init__(self,preds1,preds2,label,threshold=0.05):

'''

preds1:the output of model1

preds2:the output of model2

label :the actual label

'''

self._preds1=preds1

self._preds2=preds2

self._label=label

self.threshold=threshold

self._show_result()

def _auc(self,X, Y)->float:

return 1/(len(X)*len(Y)) * sum([self._kernel(x, y) for x in X for y in Y])

def _kernel(self,X, Y)->float:

'''

Mann-Whitney statistic

'''

return .5 if Y==X else int(Y < X)

def _structural_components(self,X, Y)->list:

V10 = [1/len(Y) * sum([self._kernel(x, y) for y in Y]) for x in X]

V01 = [1/len(X) * sum([self._kernel(x, y) for x in X]) for y in Y]

return V10, V01

def _get_S_entry(self,V_A, V_B, auc_A, auc_B)->float:

return 1/(len(V_A)-1) * sum([(a-auc_A)*(b-auc_B) for a,b in zip(V_A, V_B)])

def _z_score(self,var_A, var_B, covar_AB, auc_A, auc_B):

return (auc_A - auc_B)/((var_A + var_B - 2*covar_AB )**(.5)+ 1e-8)

def _group_preds_by_label(self,preds, actual)->list:

X = [p for (p, a) in zip(preds, actual) if a]

Y = [p for (p, a) in zip(preds, actual) if not a]

return X, Y

def _compute_z_p(self):

X_A, Y_A = self._group_preds_by_label(self._preds1, self._label)

X_B, Y_B = self._group_preds_by_label(self._preds2, self._label)

V_A10, V_A01 = self._structural_components(X_A, Y_A)

V_B10, V_B01 = self._structural_components(X_B, Y_B)

auc_A = self._auc(X_A, Y_A)

auc_B = self._auc(X_B, Y_B)

# Compute entries of covariance matrix S (covar_AB = covar_BA)

var_A = (self._get_S_entry(V_A10, V_A10, auc_A, auc_A) * 1/len(V_A10)+ self._get_S_entry(V_A01, V_A01, auc_A, auc_A) * 1/len(V_A01))

var_B = (self._get_S_entry(V_B10, V_B10, auc_B, auc_B) * 1/len(V_B10)+ self._get_S_entry(V_B01, V_B01, auc_B, auc_B) * 1/len(V_B01))

covar_AB = (self._get_S_entry(V_A10, V_B10, auc_A, auc_B) * 1/len(V_A10)+ self._get_S_entry(V_A01, V_B01, auc_A, auc_B) * 1/len(V_A01))

# Two tailed test

z = self._z_score(var_A, var_B, covar_AB, auc_A, auc_B)

p = st.norm.sf(abs(z))*2

return z,p

def _show_result(self):

z,p=self._compute_z_p()

print(f"z score = {z:.5f};\np value = {p:.5f};")

if p < self.threshold :print("There is a significant difference")

else: print("There is NO significant difference")

# Model A (random) vs. "good" model B

preds_A = np.array([.5, .5, .5, .5, .5, .5, .5, .5, .5, .5])

preds_B = np.array([.2, .5, .1, .4, .9, .8, .7, .5, .9, .8])

actual= np.array([0, 0, 0, 0, 1, 0, 1, 1, 1, 1])

DelongTest(preds_A,preds_B,actual)

# RPR与sofa比较

DelongTest(data_pred_train.rpr_15_train,data_pred_train.sofa_15_train,data_pred_train.Expire_flag15)

# RPR与sapsii比较

DelongTest(data_pred_train.rpr_15_train,data_pred_train.sapsii_15_train,data_pred_train.Expire_flag15)

# RPR与qsofa比较

DelongTest(data_pred_train.rpr_15_train,data_pred_train.qsofa_15_train,data_pred_train.Expire_flag15)

# RPR与sofa比较

DelongTest(data_pred_test.rpr_15_test,data_pred_test.sofa_15_test,data_pred_test.Expire_flag15)

# RPR与sapsii比较

DelongTest(data_pred_test.rpr_15_test,data_pred_test.sapsii_15_test,data_pred_test.Expire_flag15)

# RPR与qsofa比较

DelongTest(data_pred_test.rpr_15_test,data_pred_test.qsofa_15_test,data_pred_test.Expire_flag15)

# RPR与sofa比较

DelongTest(data_pred_train.rpr_30_train,data_pred_train.sofa_30_train,data_pred_train.Expire_flag30)

# RPR与sapsii比较

DelongTest(data_pred_train.rpr_30_train,data_pred_train.sapsii_30_train,data_pred_train.Expire_flag30)

# RPR与qsofa比较

DelongTest(data_pred_train.rpr_30_train,data_pred_train.qsofa_30_train,data_pred_train.Expire_flag30)

# RPR与sofa比较

DelongTest(data_pred_test.rpr_30_test,data_pred_test.sofa_30_test,data_pred_test.Expire_flag30)

# RPR与sapsii比较

DelongTest(data_pred_test.rpr_30_test,data_pred_test.sapsii_30_test,data_pred_test.Expire_flag30)

# RPR与qsofa比较

DelongTest(data_pred_test.rpr_30_test,data_pred_test.qsofa_30_test,data_pred_test.Expire_flag30)
